# Development of a candidate reference method for the simultaneous quantification of betaine, choline and trimethylamine N-oxide in serum samples by two-dimensional liquid chromatography and isotope dilution tandem mass spectrometry

**DOI:** 10.1007/s00216-025-05914-z

**Published:** 2025-05-23

**Authors:** Daniela Pineda-Cevallos, María Castañón Apilánez, Elena López-Cancio, Belén Prieto García, J. Ignacio García Alonso, Pablo Rodríguez-González

**Affiliations:** 1 Department of Physical and Analytical Chemistry, University of Oviedo, Avenida Julián Clavería, 8, 33006 Oviedo, Spain; 2 Department of Neurology, Central University Hospital of Asturias, 33011 Oviedo, Spain; 3 Clinical Biochemistry, Laboratory of Medicine, Central University Hospital of Asturias, 33011 Oviedo, Spain; 4 Instituto de Investigación Sanitaria del Principado de Asturias (ISPA), 33011 Oviedo, Spain

**Keywords:** Stroke biomarkers, Bidimensional chromatography, Tandem mass spectrometry, Isotope dilution

## Abstract

Serum choline, betaine and trimethylamine N-oxide levels are associated with the risk of cardiovascular events. However, no reference procedure for the determination of these compounds in serum has been developed so far. This work describes the combination of IDMS and two-dimensional liquid chromatography operating in multiple heart-cutting mode for the simultaneous quantification of the three compounds in human serum by isotope dilution tandem mass spectrometry. A reversed-phase separation is proposed as the first dimension and coupled with cation exchange chromatography in the second dimension. The online isolation of the single fraction in which the three analytes co-elute from the first dimension enables a rapid chromatographic separation in the second dimension through cation exchange. The method was validated according to the Clinical and Laboratory Standards Institute (CLSI) guidelines and applied to the analysis of 74 serum samples from patients who had suffered from an ischemic stroke in the past 24 h for further study of these metabolites as potential biomarkers to predict an ischemic stroke patient’s prognosis.

**Graphical Abstract:**

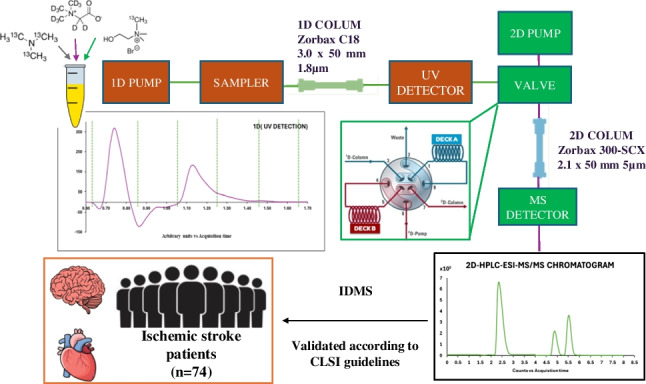

**Supplementary Information:**

The online version contains supplementary material available at 10.1007/s00216-025-05914-z.

## Introduction

Stroke is one of the worldwide leading causes of mortality and disability. Also, the risk of a further episode increases in people having experienced a stroke [1]. Risk factors include high blood pressure, tobacco use, physical inactivity, unhealthy diet, raised blood lipid levels, obesity, stress, and depression [2]. Accurate identification of novel molecular biomarkers is highly desirable for better refining risk prediction and understanding the pathogenesis of recurrent cardiovascular events after stroke.

Choline is an essential nutrient involved in neurotransmitter synthesis, cell membrane integrity, lipid transport, and methyl-group metabolism. It serves as a precursor to metabolites like betaine and trimethylamine n-oxide (TMAO) [3, 4]. Obtained from foods such as fish, meat, and eggs, choline and betaine are metabolized in the gut into trimethylamine (TMA) which is then converted to TMAO [5]. Choline deficiency is linked to organ dysfunction, stunting, and neural tube defects, while adequate levels may support memory and cardiovascular health [6]. Notably, choline, betaine, and TMAO levels are associated with cardiovascular disease risk, including stroke [5].

Recent studies have linked plasma choline pathway metabolites to both reduced and increased risks of stroke and cardiovascular events, making their role in stroke development uncertain [7, 8]. Higher dietary intake of choline was associated with a lower risk of incident ischemic stroke [9]. However, other studies associate elevated choline levels with an increased risk of recurrent stroke and unfavourable outcomes [5, 10]. A U-shaped relationship between betaine levels and ischemic stroke risk has been observed [11], while elevated TMAO levels are associated with higher stroke risk and poor prognosis [10, 12, 13]. Also, TMAO has been reported as a predictor of both poor prognosis and mortality risk in stroke patients [14]. Phosphatidylcholine is very common in some foods, such as red meat and is metabolized through the gut microbiota to choline, betaine, and TMAO. These choline metabolites have been related to the incidence of stroke and TMAO has been pointed out as a possible independent factor not only in the incidence but also in the severity and prognosis of ischemic stroke.

Mass spectrometry (MS) is increasingly used in medical laboratories due to its high selectivity and sensitivity [15, 16]. LC–ESI–MS/MS is the preferred technique for accurately quantifying clinical biomarkers [17, 18], though matrix effects in the electrospray source (ESI) can impact accuracy and precision [19, 20]. The use of isotopically labelled analogues as internal standards is widely regarded as the most efficient standardization strategy to correct for matrix effects [21]. IDMS methods are classified as higher-order measurement procedures and therefore can be considered potential candidates for reference methods [22–24]. Several reference IDMS based methods/procedures have been approved by the JCTLM for the determination of metabolites in plasma, serum and urine using GC-IDMS or LC-IDMS [25–31].

Bidimensional liquid chromatography in Multiple Heart Cutting (MHC) mode offers an effective alternative to extensive sample clean-up, minimizing matrix effects. Previously applied in our laboratory for metabolite and peptide quantification in human serum and plasma [32–34], this technique isolates target compounds by collecting fractions from the first-dimension effluent and injecting them into a second-dimension chromatography for further separation [35]. By utilizing two different stationary and/or mobile phases, MHC enhances chromatographic resolution and selectivity, successfully isolating peaks from complex matrices [36, 37]. This approach improves accuracy by reducing ion suppression caused by matrix effects [38].

This work describes the combination of IDMS and two-dimensional liquid chromatography operating in the MHC mode for the reliable and simultaneous quantification of choline, betaine and TMAO in human serum. A reversed-phase separation was selected in the first dimension to isolate the polar metabolites from potential matrix interferences and was coupled with a cation exchange chromatography in the second dimension. The online isolation of the single fraction in which the three analytes co-elute from the first dimension enabled a rapid chromatographic separation in the second dimension through cation exchange, thus reducing the matrix effects. A simple, sensitive and specific method was developed here and validated according to Clinical and Laboratory Standards Institute (CLSI) guidelines. The newly developed method was successfully applied to the analysis of 74 serum samples from patients who had suffered from an ischemic stroke in the past 24 h for further study of these metabolites as potential biomarkers to predict ischemic stroke patient prognosis.

## Experimental

### Instrumentation

An Agilent 1290 Infinity II 2D-HPLC system coupled to an Agilent 6460 triple quadrupole mass spectrometer with an electrospray jet stream source was used. OpenLab CDS Chemstation and MassHunter Acquisition software controlled the 2D-HPLC and mass spectrometer. The first dimension included a 1290 binary pump, autosampler, thermostated column compartment, and a 1260 wavelength detector, with the two dimensions connected via a dual valve with selector valves and sampling loops. The same system was used for 1D-HPLC by directly connecting the column to the MS. Sample preparation involved gravimetric standard solution preparation with an analytical balance model MS205DU (Mettler Toledo, Zurich, Switzerland), centrifugation, homogenization, and solvent removal using specialized laboratory equipment as described previously [34]. Analyte concentrations were calculated using IDMS Microsoft Excel© and IsoPatrn© software [39].

### Reagents and materials

Betaine hydrochloride, choline chloride and trimethylamine N-oxide dihydrate were purchased from Sigma-Aldrich (St. Louis, MO, USA). ^13^C_1_-labelled choline bromide (methyl-^13^C) was purchased from Sigma-Aldrich (St. Louis, MO, USA). D_11_-Betaine, D_9_-Trimethylamine N-oxide were purchased from Cambridge Isotope Laboratory (Andover, MA, USA) and Trimethylamine-^13^C_3_ N-oxide Hydrate from Toronto Research Chemicals (Toronto, Canada). Acetonitrile and Methanol (Optima ™ LC–MS Grade) was purchased from Fisher Scientific (Waltham, MA, USA). Ammonium formate was obtained from Fluka (Waltham, MA, USA). Formic acid (> 98%), and trifluoroacetic acid (99%) was purchased from Sigma-Aldrich. All solvents and reagents were of analytical reagent grade. Stock solutions of betaine, choline and TMAO were gravimetrically prepared and dissolved in ultrapure water. All solutions were kept at − 20 °C.

### Serum samples

The samples were provided by the Department of Neurology of the Central University Hospital of Asturias (HUCA). This study adheres to the tenets of the Declaration of Helsinki on Biomedical Research Involving Human Subjects, and full ethical approval was obtained from the Clinical Research Ethics Committee (Code 102/13) of the Principality of Asturias (Oviedo, Spain). These serum samples were pooled, aliquoted and stored at − 80 °C until the analyses. A total number of 74 patient serum samples were analysed with the optimized method.

### Procedures

#### Sample treatment

A gravimetrically controlled 0.2 g of serum and approximately 0.065 g of an isotopically labelled analogue mixture containing betaine, choline, and TMAO were added to a 1.5-mL Eppendorf tube. The mixture was vortexed for 1 min for homogenization, followed by the addition of 300 µL methanol for protein precipitation. After vortex mixing, the solution was centrifuged at 13,000 rpm for 10 min to remove proteins. The supernatant was then transferred, evaporated to dryness, reconstituted with 0.1% TFA in water, and filtered through a 0.2-μm PVDF syringe filter before injection into the 2D-LC–MS/MS system.

#### Measurements by 2D-HPLC–ESI–MS/MS

**Fig. 1:**
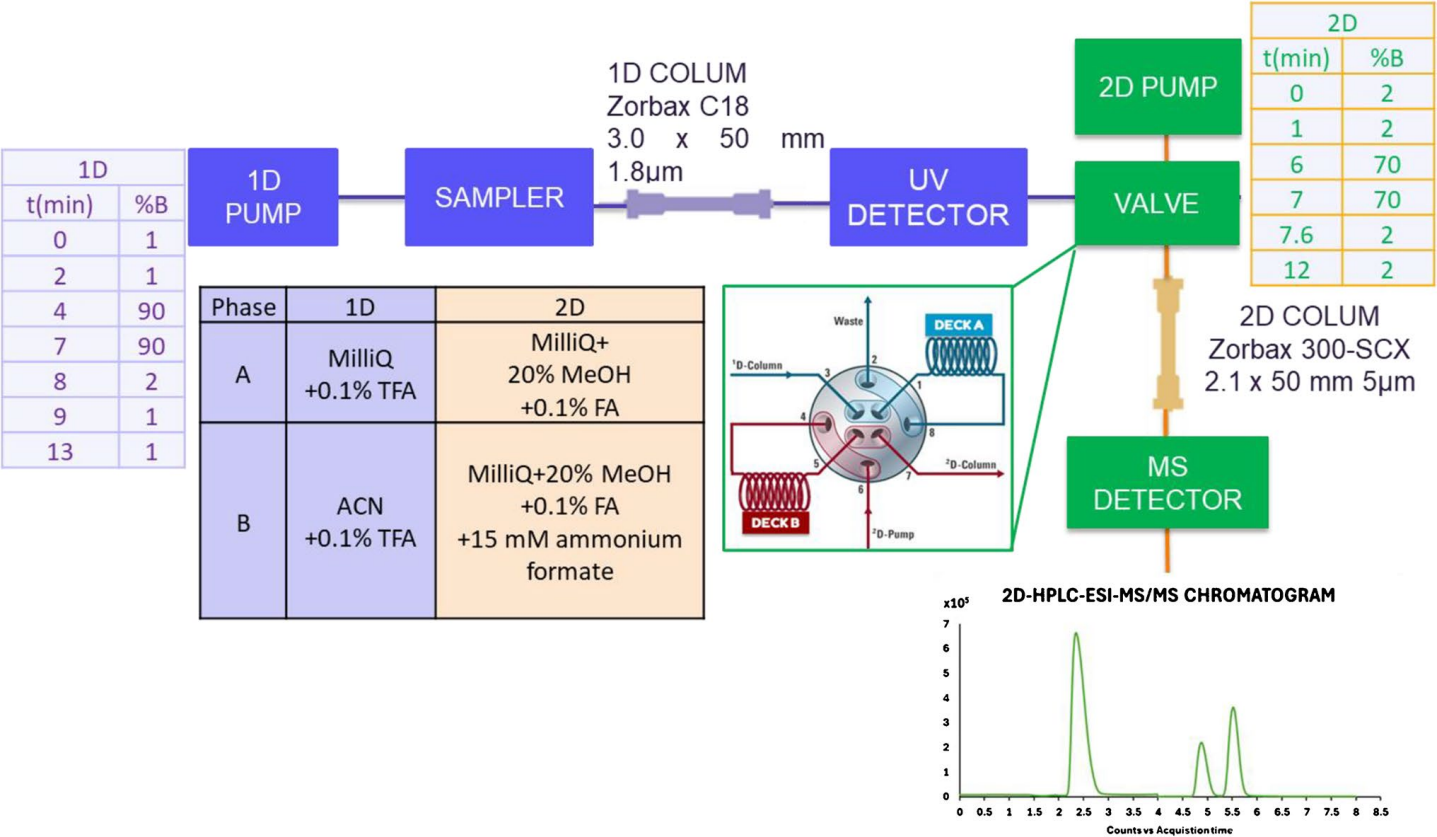
Instrumental set-up used in this work for the quantification of choline, betaine and TMAO by 2D-LC–ESI–MS/MS

Figure 1 shows the chromatographic conditions applied in this work. The first-dimension (1D) chromatographic separation was performed using a ZORBAX Eclipse Plus C18 column (3.0 × 50 mm, 1.8 µm) at 35 °C. The mobile phases were 0.1% TFA in ultrapure water (A) and acetonitrile (B), with a 10-µL injection volume and a 0.4 mL/min flow rate. The second-dimension (2D) separation used a Zorbax 300-SCX column (2.1 × 50 mm, 5 µm) at 35 °C. A fraction of 80 µL from the 1D mobile phase was stored and transferred. The mobile phases were 0.1% formic acid with 20% methanol in water (A) and 0.1% formic acid, 20% methanol, and 15 mM ammonium formate in water (B). The total run time was 14 min. The second dimension incorporated also a 1290 Infinity binary pump and a 2-pos/4-port duo valve connected the two dimensions and was coupled to two selector valves that included six 80 µL sampling loops per valve (Agilent Technologies). Taking into account the co-elution and low retention time of the targeted metabolites in the C18 column, a fraction of 80 µL of the mobile phase of the fist dimension was stored in a sampling loop at 0.84 min and then it was transferred to the second dimension. The 2D-HPLC system was coupled to a triple quadrupole mass spectrometer equipped with an electrospray source working in positive ionization mode.

The optimized conditions of the ionization source are given in Table S1 of the supporting information. For all compounds, the protonated molecular ion [M + H]^+^ was selected as precursor ion and all samples were measured using the Multiple Reaction Monitoring mode (MRM). Fragmentation of the precursor ions by Collision Induced Dissociation (CID) was carried out with nitrogen as collision gas. The acquired MRM transitions for natural and labelled analogues are given in Table S2. The fragmentor voltage was set at 110 V, 70 V, and 60 V for betaine, choline and TMAO, respectively. Optimized collision energy was set at 30, 18 and 20 V, for betaine, choline and TMAO, respectively.

#### Quantification of betaine, choline and TMAO by IDMS

The analyte concentrations were determined by IDMS combined with multiple linear regression. The theoretical isotope distribution of both natural and labelled fragment ions was calculated using IsoPatrn© software [39]. In this approach, the experimental isotopologue distribution of a given product ion in the sample (*A*^*m*^) is expressed as a linear combination of the natural abundance product ion (*A*^*nat*^) and the isotopically labelled analogues (*A*^*lab*^). This isotopologue distribution is then deconvoluted using multiple linear regression, following Eq. (1) for n isotopologues*.*

1 $$\left[\begin{array}{c}{A}_{1}^{m}\\ \begin{array}{c}{A}_{2}^{m}\\ {A}_{3}^{m}\end{array}\\ {A}_{n}^{m}\end{array}\right]=\left[\begin{array}{c}{A}_{1}^{nat}\\ \begin{array}{c}{A}_{2}^{nat}\\ {A}_{3}^{nat}\end{array}\\ {A}_{n}^{nat}\end{array} \begin{array}{c}{A}_{1}^{lab}\\ \begin{array}{c}{A}_{2}^{lab}\\ {A}_{3}^{lab}\end{array}\\ {A}_{n}^{lab}\end{array}\right]\times \left[\begin{array}{c}{X}_{nat}\\ {X}_{lab}\end{array}\right]+\left[\begin{array}{c}{e}_{1}\\ \begin{array}{c}{e}_{2}\\ {e}_{3}\end{array}\\ {e}_{n}\end{array}\right]$$

In Eq. (1) A^m^, A^nat^, and A^lab^ are the relative abundances for four selected MRM transitions for the mixture (m), the sample (nat) and the tracers (lab) and e refers to the error vector. The unknowns *X*_*nat*_ and *X*_*lab*_ are the molar fractions of sample and tracer in the mixture respectively and can be calculated applying Eq. (1) for each compound.

The concentration of the analytes in the sample (C_nat_) was then calculated by applying Eq. (2):

2 $${C}_{nat}={C}_{lab}\times \frac{{X}_{nat}}{{X}_{lab}}\times \frac{{m}_{lab}}{{m}_{nat}}\times \frac{{w}_{nat}}{{w}_{lab}}$$

where C_lab_ is the concentration of the analogues, m_lab_ and m_nat_ are the weights taken from the labelled standard and sample and w_nat_ and w_lab_ are the molecular weights of natural abundance and labelled analytes.

## Results and discussion

### Characterization of the isotopically labelled analogues

The measurement of isotopologue distributions of natural and labelled analogues by MS/MS must be validated comparing the theoretical values obtained by IsoPatrn© [39]. Figure S1 shows that the experimental isotopologue distributions obtained for both natural and labelled analogues agreed well with the theoretical values. The isotopic enrichment of the labelled analogues was calculated as described previously [40] obtaining 99.6 ± 0.1%, 99.8 ± 0.1%, 99.48 ± 0.1% and 99.92 ± 0.1% for ^13^C_1_-choline, D_11_-betaine, ^13^C_3_-TMAO and D_9_-TMAO, respectively. Finally, the concentration of the working solutions of the labelled compounds was calculated by reverse isotope dilution using natural abundance standards.

### Evaluation of TMAO dimer formation and its suitability for IDMS

To carry out the IDMS quantification by tandem mass spectrometry, it is necessary to select optimal precursor and product ions. Typically, the most intense non-interfered peak in the mass spectrum is selected. Figure S2 shows the mass spectra for natural abundance and labelled D_9_-TMAO obtained in SCAN mode in which the most intense m/z values were 151 and 169, respectively, which correspond to the TMAO dimerization in the ESI source. Initial IDMS optimization studies were carried out using these most intense ions as precursor ions which showed to be inadequate. Figure S3A shows that the ratio of natural to labelled analogue dimerization varies with the molar fraction of the labelled analogue (0.1, 0.5 and 0.9) and a mixed dimer of mass 160 is also formed. When the D_9_-TMAO molar fraction is 0.1 the formation of the natural dimer is favoured while the opposite occurs for a molar fraction of 0.9. As can be observed, the formation of the mixed dimer m/z 160 also occurs and is the most abundant at equimolar fractions. This phenomenon fits a binomial distribution as described in Fig. S3B showing which dimer is formed depending on the molar fraction of the labelled compound. For IDMS quantification the measurement of the dimer has clear negative consequences: at low molar fraction of the labelled analogue the natural dimer is mostly formed and TMAO quantification is overestimated. On the other hand, when the molar fraction of the labelled compound is high, the formation of the labelled dimer is favoured, leading to an underestimation of the analyte. Accurate quantification only occurs with equimolar fractions, resulting in the formation of, mostly, the mixed dimer.

In any case, equimolar fractions cannot be ensured during the analysis of serum samples so working with the TMAO monomer instead of the dimer is mandatory despite its lower sensitivity. To demonstrate this assumption, we analysed standard solutions at three different levels adding the same amount of the labelled analogue (molar fraction of the labelled analogue of 0.7, 0.2 and 0.1). The analyses were performed using D_9_-TMAO and ^13^C_3_-TMAO as labelled analogues. The SRM transitions corresponding to the TMAO monomer and those of the TMAO dimer formed during ESI ionization were monitored and *n* = 5 independent injections were carried out. The results obtained are summarized in Table S3 and show that when working with the dimer and a molar fraction of 0.70 a recovery of about 20% is obtained whereas when the molar fraction is 0.1 the recovery obtained increases to 700%. However, when monitoring the SRM transitions of the monomer both using the deuterated or ^13^C-labelled monomer recoveries close to 100% were obtained at all concentration levels.

### Development of the 2D-HPLC–MS/MS methodology

The determination of choline, betaine and TMAO by LC–ESI–MS/MS can be carried out using strong cation exchange chromatography (SCX). However, there are two limitations when SCX chromatography is directly coupled to the ESI for the analysis of complex matrices like human serum. First, spectral interferences at the analyte’s retention time in serum samples due to matrix constituents are likely to occur even when using MS/MS strategies. Secondly, the sample throughput of the SCX column is significantly compromised due to its lower matrix tolerance compared to that of a reversed-phase (C18) column [41].

To avoid these problems, we propose here a 2D-LC strategy based on the use of a reversed-phase chromatography C18 in the first dimension and a SCX chromatography in the second dimension. Highly polar compounds like choline, betaine and TMAO will not show retention in the C18 column but can be simultaneously transferred in a single fraction to the second dimension in which they will be separated by SCX. In this way, we obtain four potential advantages: (i) minimization of the ionization suppression and spectral interferences due to matrix constituents, (ii) simultaneous analyte enrichment and sample purification, (iii) enhancement of the SCX column life due to a lower introduction of matrix content and (iv) the use of mobile phases not compatible with ESI ionization (like the use of TFA as organic modifier) in the first dimension to enhance retention of interfering compounds in the reversed-phase chromatography.

#### Selection of the 1D mobile phase

**Fig. 2:**
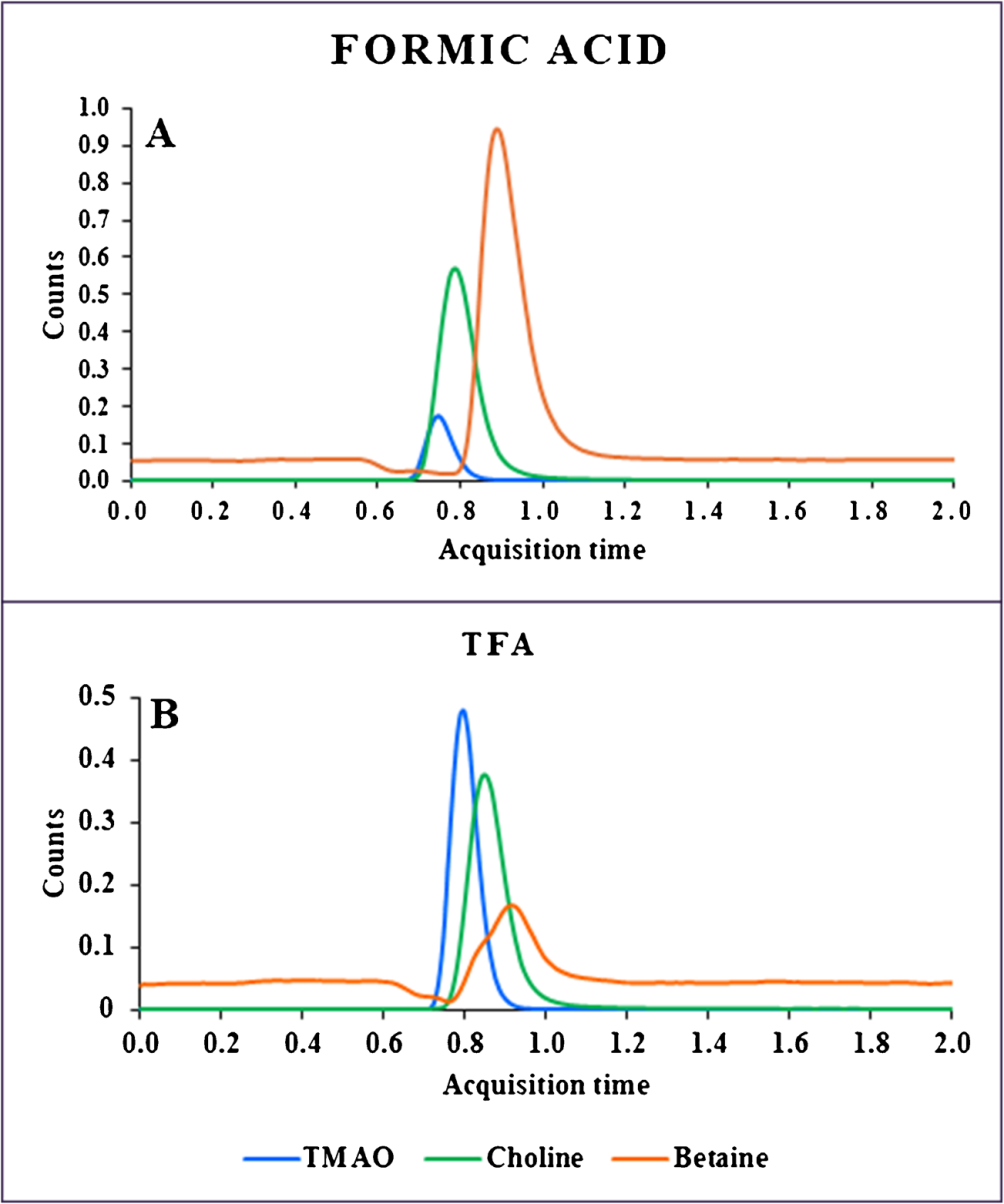
Reverse phase 1D-HPLC–MS/MS chromatogram of a mixture of choline, betaine and TMAO applying the same chromatographic conditions but using **A** 0.1% formic acid as mobile phase A and acetonitrile as mobile phase B and **B** 0.1% TFA as mobile phase A and acetonitrile as mobile phase B

When optimizing the MHC strategy, the first step is the selection of chromatographic conditions in the first dimension. The aim is to ensure that choline, betaine and TMAO co-elute in a narrow band so that they can be simultaneously transferred to the second dimension in a single fraction. To do so, we analysed a standard mixture of the three compounds coupling the SCX column directly to the ESI–MS/MS system. We compared two different organic modifiers: formic acid (Fig. 2A) and TFA (Fig. 2B) using the chromatographic conditions described in the Experimental section. The use of formic acid improved peak shape for betaine but TFA provided a higher co-elution of the three analytes. Also, TFA provided higher sensitivity for TMAO which is particularly important due to the low concentration of this analyte in human serum and the previously described dimerization [42]. For these reasons, TFA was selected as modifier for the further analysis of serum samples.

#### Optimization of the time window and loop volume for the transfer of the 1D heart-cuts

**Fig. 3:**
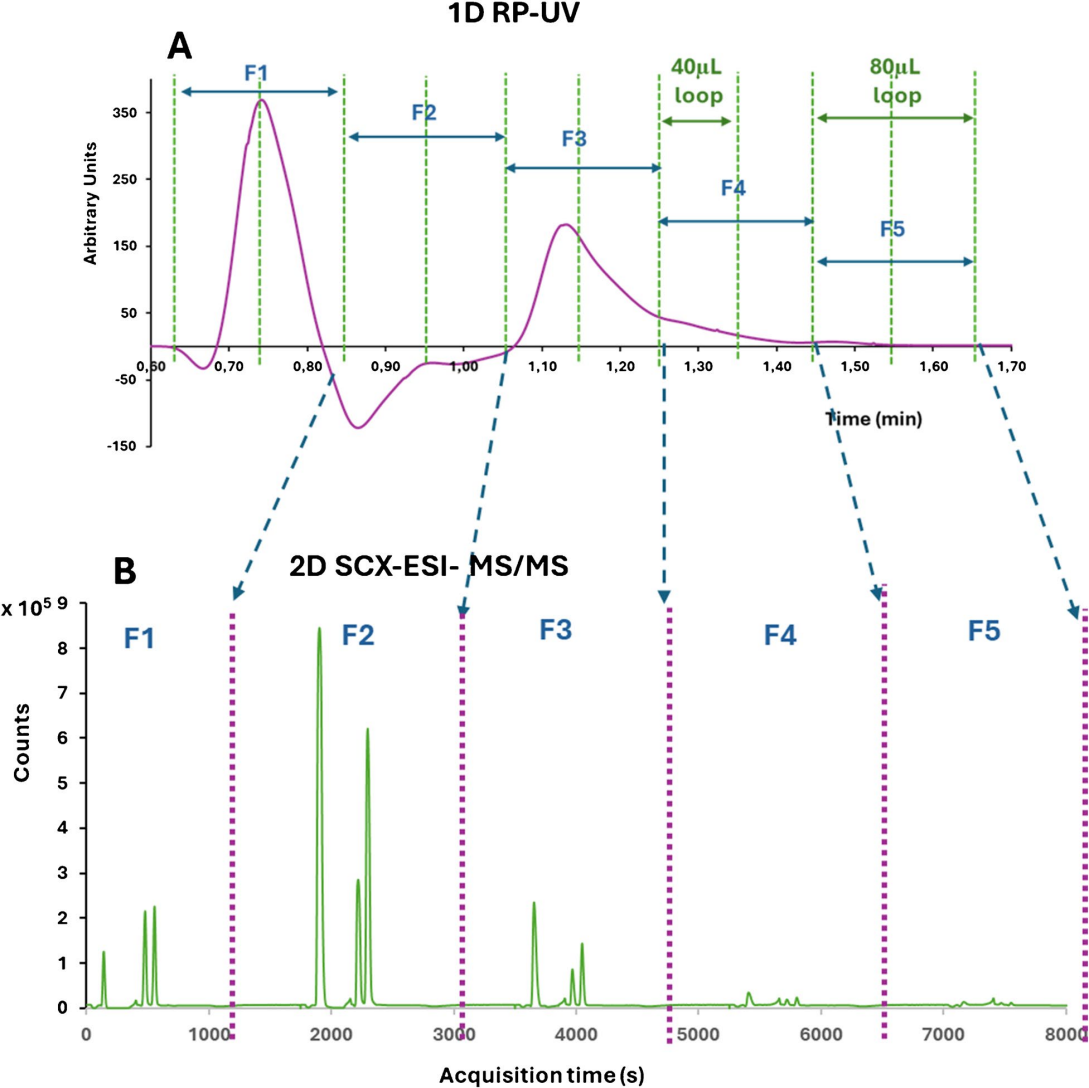
**A** 1D-LC-UV chromatogram of a pooled serum sample obtained by reversed-phase chromatography using a C18 column. **B** 2D-LC–ESI–MS/MS chromatograms of five consecutive 80 μL heart-cuts (F1, F2, F3, F4 and F5) using strong cation exchange chromatography

Figure 3A shows the LC-UV chromatogram of a pooled serum sample analysed using a C18 column under the specified chromatographic conditions with TFA as the organic modifier. Due to the UV detector’s lack of selectivity, choline, betaine, and TMAO retention times could not be identified, as interfering matrix components affected absorbance and baseline stability. Consequently, the LC-UV chromatogram could not be used to select fractions for the second dimension. Two loop volumes (40 µL and 80 µL) were used, transferring five consecutive heart-cuts to the second dimension over 6 or 12 s, respectively. The strategy relies on the co-elution of analytes and their isotopically labelled analogues. If co-elution occurs, full transfer to the second dimension is unnecessary, preserving mass balance. However, if a chromatographic isotope effect arises, complete transfer of both analogues is required to maintain accuracy.

Figure 3B shows the chromatograms for five consecutive 80 μL fractions, demonstrating the high-resolution sampling strategy that enables the transfer of multiple heart-cuts to the second dimension. Each fraction is analysed sequentially by SCX chromatography and MS/MS detection to determine which fraction contains the highest concentration of analytes. The chromatograms indicate that Fraction 2 (F2) appears to be the most favourable, though all fractions contain detectable levels of the three compounds. A challenge in this analysis is assessing which fraction maintains isotopic equilibrium between the natural and labelled compounds, as chromatographic isotope effects—especially for deuterium-labelled analytes—could occur. To evaluate the presence of these isotopic effects, IDMS calculations are applied to the different extracted fractions.

Figure S4 shows the concentrations obtained by IDMS for choline, betaine and TMAO in a pooled serum sample analysed by high-resolution sampling. ^13^C_1_-labelled choline, D_11_-betaine and D_9_-TMAO were used as labelled analogues and five consecutive heart-cuts were transferred to the second dimension. This experiment was performed both with 40 and 80 µL loops for comparison. For choline no clear differences were found between 40 and 80 μL loops while for betaine and TMAO much higher concentrations were obtained with the 40 μL loops which, we think, is related to the use of deuterated compounds as tracers. For betaine and TMAO, concentrations up to 16 and 4 μg g^−1^, respectively, were obtained using 40 µL loops. Considering that the maximum endogenous concentrations reported for betaine and TMAO in serum are 5.22 µg g^−1^ and 0.83 μg g^−1^, respectively [43, 44], it can be concluded that using 40 μL loops mass balances are not maintained leading to a significant overestimation of the concentration of both compounds in serum. According to this a higher amount of the analyte was transferred compared to the labelled analogue due to a chromatographic isotope effect between the deuterated analogues and the analytes as demonstrated in Fig. S5 which shows the peak profiles of the three analytes and their labelled analogues obtained in the first dimension. For this experiment a new ^13^C_3_-TMAO standard was acquired to avoid chromatographic isotope effects (Fig. S5 A and D). However, slight differences can be observed for betaine and TMAO when using the multideuterated analogues D_11_-Betaine (Fig. S5 B) and D_9_-TMAO (Fig. S5 C). The difference between the retention times was 1.08 s for betaine and its deuterated analogue and 0.66 s for TMAO and its D_9_-labelled analogue. Higher loop volumes would increase the probability of maintaining the mass balance between the analyte and the labelled analogue when small differences in retention times occur so the 80 μL loops were selected for further studies. The difference between the analyte and labelled analogue retention times demonstrate the occurrence of a chromatographic isotopic effect. Other isotope effects may occur during sample preparation when using deuterated analogues leading to a broader implication in the IDMS results. However, such isotope effects can only be demonstrated comparing the accuracy and precision obtained with ^13^C and deuterated analogues.

To confirm these results, we analysed a standard mixture containing a known concentration of each analyte and a pooled serum (N0) that was diluted (N3) and also fortified (N7). The theoretical concentration levels are also given in Table 1. For both the standard and the pooled serum samples five different fractions of 80 μL were transferred to the second dimension and the concentration in each fraction was quantified by IDMS. Table 1 shows the concentration and recovery values obtained for each fraction. Consistent and satisfactory recover values were obtained for both choline and TMAO when using the ^13^C-labelled analogues. For choline recoveries from 89 to 115% were obtained in all fractions of the standard and the serum samples whereas for TMAO recovery values ranged from 94 to 105%, except for the fifth fraction of the low concentrated serum. In contrast, for betaine using a D_9_-labelled analogue recoveries ranged from 35 to 204% and changed drastically between the different fractions collected. Only the second fraction provided recoveries for betaine from 104 to 108% in all samples. In conclusion, the accuracy and precision of the proposed methodology for serum samples are influenced by the selected time window, particularly when chromatographic isotope effects occur between analytes and their labelled analogues. The second fraction, which contained the highest concentrations of the three compounds (Fig. 3B), was selected for further experiments. This fraction provided recovery values ranging from 96 to 108%.

**Table 1:** Concentrations for choline, betaine and TMAO in five consecutive 80 µL heart-cuts (F1 to F5) transferred to the second dimension when analysing a standard sample and a pooled serum sample at three concentration levels N3 (low) N0 (endogenous) and N7 (high). Concentrations in each fraction were obtained by IDMS using ^13^C_1_-choline ^13^C_3_- TMAO and D_11_-betaine. The recovery of the results in each fraction is expressed as %

	Concentration (µg g^−1^)				%Recovery			
Choline	N3	N0	N7	Standard	N3	N0	N7	Standard
F1	0.36	3.7	6.9	2.02	109	106	109	96
F2	0.36	3.7	7.0	2.03	108	107	110	97
F3	0.36	3.7	6.8	2.00	109	107	108	95
F4	0.37	3.7	6.9	1.96	111	106	109	93
F5	0.38	3.5	6.9	1.87	115	101	109	89
Theoretical	0.33 ± 0.01	3.5 ± 0.2	6.3 ± 0.3	2.10 ± 0.07				
Betaine	Concentration (µg g^−1^)				%Recovery			
F1	0.13	3.28	6.60	3.41	35	86	90	106
F2	0.40	3.95	7.90	3.39	107	104	108	106
F3	0.52	3.87	7.76	3.39	140	101	106	106
F4	0.28	3.19	6.86	4.22	74	84	94	131
F5	0.24	2.39	6.18	6.55	64	63	84	204
Theoretical	0.37 ± 0.01	3.82 ± 0.2	7.34 ± 0.4	3.21 ± 0.01				
TMAO	Concentration (µg g^−1^)				%Recovery			
F1	0.106	1.102	2.16	0.857	97	101	104	101
F2	0.105	1.096	2.17	0.851	96	101	105	100
F3	0.107	1.109	2.16	0.856	98	102	104	101
F4	0.103	1.105	2.17	0.853	94	102	105	100
F5	0.039	1.110	2.17	0.0856	35	102	105	101
Theoretical	0.11 ± 0.01	1.09 ± 0.01	2.07 ± 0.01	0.85 ± 0.01				

#### Optimization of the injection volume

**Fig. 4:**
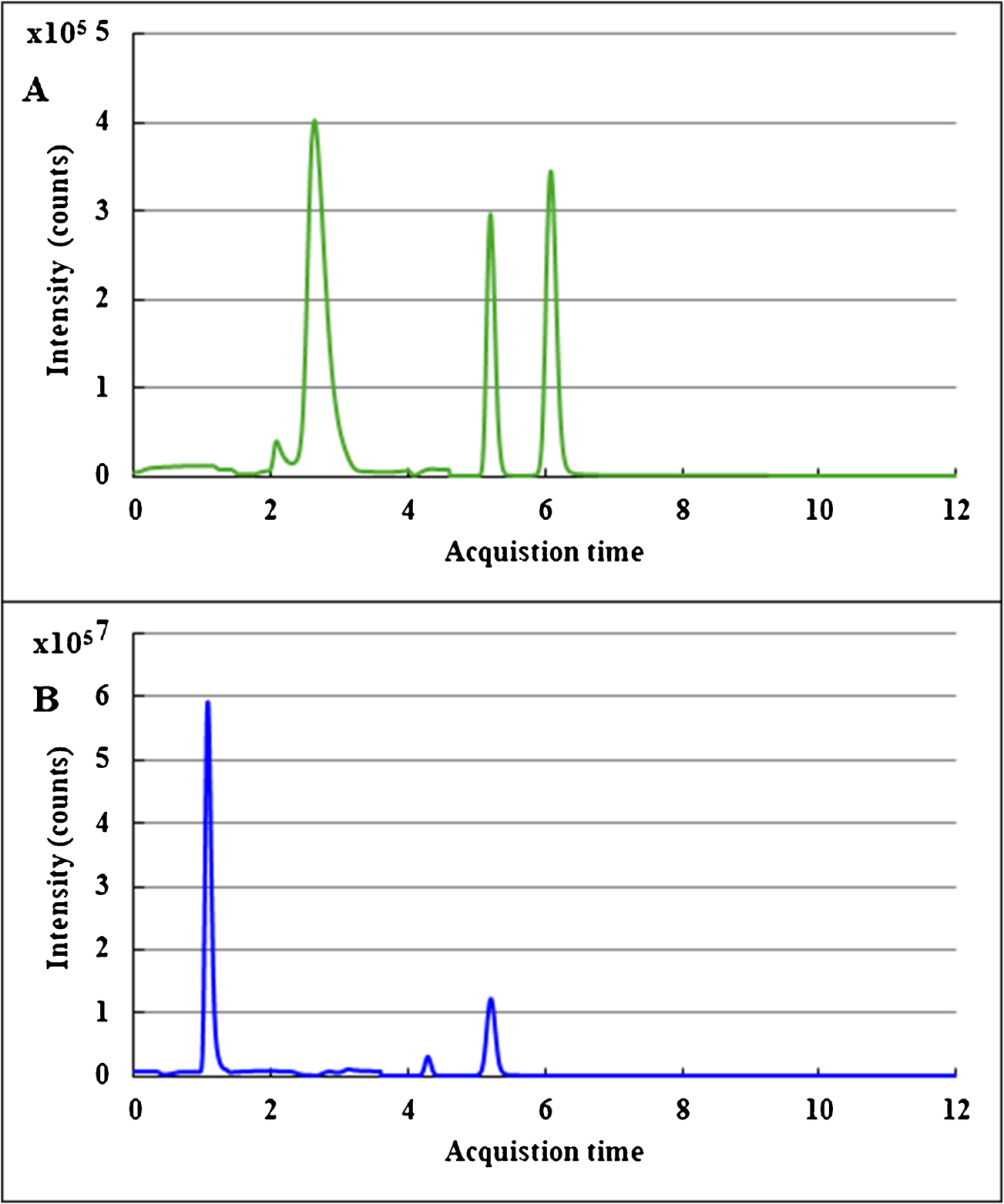
**A** 1D-LC-ESI-MSMS chromatogram of a serum sample using strong cation exchange chromatography. **B** 2D-LC–ESI–MS/MS chromatograms of the same serum samples using strong cation exchange chromatography after a previous separation the first dimension using a C18 column

Besides the increase of the SCX column life the use of the proposed 2D-LC strategy provides an additional advantage. When comparing the sensitivity obtained by 1D and 2D methodologies an increase in the peak area of 2-, 14- and threefold was observed for betaine, TMAO and choline, respectively, as shown in Fig. 4. This demonstrates an efficient removal of most of the matrix constituents that affects the ionization particularly for TMAO using the 2D strategy. The injection volume was then optimized to obtain the highest sensitivity without increasing ionization suppression. To do so, the second fraction of a pooled serum sample was measured using seven different injection volumes (0.5, 1, 2, 3, 5, 10 and 15 μL). Figure S6 confirms the negligible presence of matrix in the transferred heart-cuts, as ionization suppression is barely observed for all compounds when increasing the injection volume up to 15 μL. Finally, 10 μL was selected instead of 15 μL as the optimal injection volume to keep a better maintenance of the C18 column used in the first dimension. At this point we were ready to perform a full validation of the 2D-HPLC–MS/MS method following the guidelines of the Clinical and Laboratory Standards Institute (CLSI) guidelines.

### Validation of the 2D-HPLC–MS/MS method according to the CLSI guidelines

#### Linearity assessment of serum betaine, choline and TMAO

Linearity assessment of serum choline, betaine and TMAO determination by 2D-HPLC–ESI–MS/MS was carried out according to the CLSI EP06-A guidelines. Nine concentration levels of each analyte were measured in the same fortified or diluted pooled serum in concentrations ranging from 0.18 to 7.80, 0.19 to 8.92, and 0.059 to 2.63 µg g^−1^ for choline, betaine and TMAO, respectively. A fixed amount of labelled analogue was added to all concentration levels. The endogenous level (N0) was obtained from the direct measurement of the serum pool, whereas higher concentration levels (N6, N7 and N8) were prepared by adding known amounts of natural standards to the serum pool. Concentrations below the endogenous level (N1 to N5) were obtained by diluting the pooled serum with phosphate buffered saline (PBS). For each concentration level, five replicates were injected into the 2D-HPLC–ESI–MS/MS. Table S4 shows, for each level, the analyte concentration and the added amounts of labelled compounds and Fig. S7 shows the results when plotting the theoretical concentration versus the experimental concentration. As can be observed a satisfactory linearity was obtained within the studied range for the three compounds. The correlation coefficients obtained were 0.9998, 0.9999, and 0.9999 for betaine, choline, and TMAO, respectively.

#### Trueness of the methodology

The trueness of the 2D-HPLC–MS/MS methodology was evaluated following the CLSI C62 guidelines. Due to the absence of reference materials and reference methods we performed spike and recovery analyses of a diluted and fortified pooled serum. In each measurement session, the endogenous concentration of the target compounds in the pooled serum was determined by analysing five independent replicates by 2D-HPLC–MS/MS. Fortified samples (N6, N7 and N8) were prepared by adding to 0.20 µL of the pooled serum, known amounts of natural abundance analytes. Concentrations below the endogenous level (N1 and N3) were obtained by diluting the serum pool with phosphate buffered saline (PBS). To all samples, a known amount of the three labelled analogues was added for IDMS quantification. The whole experiment was repeated in six different measurement sessions to evaluate the reproducibility of the recoveries. The concentration range studied was therefore between 0.17–8.17, 0.22–9.21, and 0.06–2.59 µg g⁻^1^ for choline, betaine, and TMAO, respectively. Tables S5, S6 and S7 show the individual results obtained for choline, betaine and TMAO, respectively. Table 2 shows the results obtained when plotting the added concentration vs. the experimental concentration of all the individual measurements carried out. The intercept values represent the endogenous levels of the analytes in the pooled serum whereas the slope × 100 represents the global recoveries (%). Table 2 shows that satisfactory recoveries of 100.5 ± 0.5%, 99.8 ± 0.4% and 100.9 ± 0.1%, were obtained for choline, betaine and TMAO respectively.

**Table 2:** Slope × 100 (% recovery), intercept (endogenous concentration) and correlation coefficient when plotting the theoretical concentrations versus the experimental concentrations obtained for choline, betaine and TMAO in a pooled serum by 2D-HPLC–ESI–MS/MS. Uncertainty of the values corresponds to the standard deviation obtained from *n* = 5 recovery experiments carried out in six different days. Each recovery experiment was carried out at five different concentration levels

	Choline	Betaine	TMAO
Correlation coefficient R^2^	0.9994	0.9996	1.0000
Endogenous concentration (µg g^−1^) (intercept)	3.5 ± 0.2	3.73 ± 0.07	1.09 ± 0.01
% Recovery (slope × 100)	100.5 ± 0.5	99.8 ± 0.4	100.9 ± 0.1

#### Intra-day and inter-day variability

The intra-day and inter-day variabilities were evaluated following the CLSI EP15-A2 guidelines in six different days. In each day, at least 5 replicates of a diluted (N1 and N3) and fortified pool serum (N6, N7 and N8) were measured by 2D-HPLC–MS/MS. The results are given in Table 3. The repeatability values ranged, depending on the concentration level, from 0.15 to 1.03, from 0.14 to 3.71 and from 0.13 to 1.23 (expressed as % CV) for choline, betaine and TMAO, respectively. The reproducibility values obtained from the measurements of all sessions ranged from 4.3 to 6.4, from 3.7 to 5.8 and from 0.45 to 3.75 (expressed as %CV) for choline, betaine and TMAO, respectively.

**Table 3:** Concentration (µg^−1^) and % CV of choline, betaine and TMAO at five concentration levels. Uncertainty of the values corresponds to the standard deviation of the concentration obtained for the indicated replicates

Level	Measurement day	Concentration (µg^−1^) *n* = 5			% CV		
		Choline	Betaine	TMAO	Choline	Betaine	TMAO
N1	1	0.1838 ± 0.0012	0.211 ± 0.002	0.0588 ± 0.0001	0.65	0.76	0.24
	2	0.1775 ± 0.0005	0.191 ± 0.001	0.0562 ± 0.0003	0.31	0.37	1.23
	3	0.1687 ± 0.0004	0.190 ± 0.001	0.0578 ± 0.0002	0.24	0.69	0.15
	4	0.1962 ± 0.0003	0.181 ± 0.000	0.0579 ± 0.0006	0.50	0.90	0.16
	5	0.1687 ± 0.0004	0.189 ± 0.000	0.0574 ± 0.0006	0.45	1.28	0.29
	6	0.1775 ± 0.0005	0.191 ± 0.001	0.0562 ± 0.0003	1.03	0.75	0.52
	**Average (*n* = 30)**	**0.18 ± 0.01**	**0.19 ± 0.01**	**0.058 ± 0.001**	**6.37**	**5.80**	**1.68**
N3	1	0.329 ± 0.001	0.390 ± 0.001	0.109 ± 0.00	0.41	0.27	1.01
	2	0.347 ± 0.001	0.365 ± 0.001	0.115 ± 0.001	0.19	0.26	0.27
	3	0.315 ± 0.002	0.381 ± 0.004	0.115 ± 0.001	0.49	1.34	0.18
	4	0.321 ± 0.000	0.350 ± 0.002	0.107 ± 0.001	0.59	3.71	0.32
	5	0.322 ± 0.001	0.373 ± 0.001	0.107 ± 0.000	0.37	0.37	0.27
	6	0.349 ± 0.001	0.370 ± 0.002	0.105 ± 0.000	0.53	1.13	0.47
	**Average (*n* = 30)**	**0.33 ± 0.01**	**0.37 ± 0.01**	**0.110 ± 0.004**	**4.30**	**3.70**	**3.75**
N6	1	4.05 ± 0.01	5.14 ± 0.04	1.348 ± 0.002	0.42	0.74	0.18
	2	4.17 ± 0.01	4.49 ± 0.01	1.341 ± 0.004	0.24	0.21	0.15
	3	4.06 ± 0.02	4.72 ± 0.03	1.346 ± 0.002	0.51	0.23	0.13
	4	4.11 ± 0.01	4.49 ± 0.01	1.349 ± 0.003	0.16	0.25	0.95
	5	4.15 ± 0.01	4.66 ± 0.05	1.350 ± 0.002	0.15	0.50	0.50
	6	4.63 ± 0.01	4.57 ± 0.02	1.334 ± 0.003	0.16	0.18	0.20
	**Average (*n* = 30)**	**4.20 ± 0.22**	**4.68 ± 0.24**	**1.345 ± 0.006**	**5.23**	**5.23**	**0.45**
N7	1	6.21 ± 0.03	8.05 ± 0.07	2.087 ± 0.003	0.23	0.14	0.22
	2	6.35 ± 0.03	7.18 ± 0.10	2.074 ± 0.004	0.29	1.51	0.25
	3	6.17 ± 0.01	7.32 ± 0.02	2.068 ± 0.003	0.23	0.17	1.03
	4	6.34 ± 0.01	7.03 ± 0.01	2.075 ± 0.005	0.45	0.40	0.28
	5	6.04 ± 0.02	7.28 ± 0.04	2.084 ± 0.004	0.27	0.97	0.16
	6	6.89 ± 0.01	7.17 ± 0.03	2.053 ± 0.003	0.30	0.51	0.21
	**Average (*n* = 30)**	**6.33 ± 0.29**	**7.34 ± 0.36**	**2.07 ± 0.01**	**4.65**	**4.96**	**0.59**
N8	1	7.80 ± 0.03	9.98 ± 0.13	2.626 ± 0.008	0.58	1.20	0.19
	2	7.97 ± 0.05	9.13 ± 0.34	2.622 ± 0.008	0.28	0.70	0.58
	3	7.70 ± 0.04	9.48 ± 0.02	2.630 ± 0.003	0.42	0.41	0.51
	4	7.78 ± 0.02	9.08 ± 0.14	2.623 ± 0.006	0.15	0.49	0.22
	5	7.63 ± 0.04	9.52 ± 0.11	2.639 ± 0.005	0.20	0.40	0.13
	**Average (*n* = 30)**	**7.91 ± 0.36**	**9.35 ± 0.39**	**2.62 ± 0.02**	**4.50**	**4.13**	**0.58**

#### Blank, detection and quantification limits of the method

Blank, detection and quantification limits were calculated according to the EP17-A CLSI guidelines. First, five independent replicates of PBS performing *n* = 12 injections per blank (B) were analysed in five different days to calculate the limit of blank (LoB) according to Eq. (3).

3 $$LoB={average}_{B}+1.645{SD}_{B}$$

Secondly, 6 independent replicates of a 1:20 dilution of a pooled serum with PBS were analysed by performing *n* = 12 injections per diluted pooled plasma (low concentration sample, LCS). The detection limit (LoD) was calculated according to Eq. (4) and quantification limit was established as 10 times the standard deviation of blank measurements.

4 $$LoD=LoB+1.645{SD}_{LCS}$$

Table S8 shows that quantification limits obtained analysing the diluted pooled plasma were comparable to those obtained with PBS, and hence, no significant matrix effect was appreciated. Finally, Fig. S8 shows a representative 2D-HPLC–ESI–MS/MS chromatogram of the 1:20 dilution of the pooled serum.

#### Quality control

Intra-day and inter-day variabilities were evaluated by analysing quality control samples during the analysis of 74 real samples. The evaluation was carried out in six different days. In each day QC sample was injected 3 times at different points of the measurement session. The results are given in Table S9. The intra-day precision expressed as CV (%) ranged from 0.08 to 0.11%, from 0.61 to 3.91% and from 0.02 to 0.92% for choline, betaine and TMAO, respectively. Inter-day precision was determined as 6.43% for choline, 7.53% for betaine and 1.88% for TMAO.

#### Carryover evaluation

Carryover was evaluated by injecting low concentrated samples of 0.18, 0.19 and 0.06 µg g^−1^ after 50-fold more concentrated samples, 7.78, 9.24 and 2.62 µg g^−1^ for choline, betaine and TMAO, respectively. These results were compared with those obtained from the measurement of 10 sequential replicates. Fischer’s test rejected the equality of the variances of the sample sets (F > F_critical_). Therefore, the independent samples t-test was employed assuming unequal variances and showed that there were no significant differences (t _stat_ < t_critical_) between sequentially measured samples and those measured after high concentration samples. Table S10 summarizes the results of Fischer and t-test. Also, we evaluated the carryover by injecting PBS after the measurement of high concentrated samples, Fig. S9 shows a chromatogram of a high concentration serum sample vs a chromatogram of PBS injected after the high concentrated sample. No detectable signal of the analytes was observed for the concentration range of the samples analysed in this work: from 0.18 to 7.78 µg g^−1^ for choline, from 0.19 to 9.24 µg g^−1^ for betaine and 0.06 to 2.62 µg g^−1^ for TMAO.

#### Stability of the serum samples

Three different storage conditions of the sample were evaluated analysing aliquots of a high concentrated and a low concentrated pooled serum sample of known concentration after the storage time. The first condition evaluated was 8 h in the HPLC autosampler at 10 °C. Secondly, bench-top stability was tested by analysing the serum samples after leaving the samples for 24 h at room temperature (25 °C). In addition, stability of the serum samples was evaluated after three freeze and thaw cycles (from − 20 °C to room temperature). Table 4 summarizes the results obtained for the three storage conditions for the high and low concentrated pooled serum samples. The results indicated that the analytes were stable under all these conditions. Recovery values were calculated from the ratio of the concentration obtained after and before the storage period. All recoveries ranged from 93 to 114% These results showed that the analytes were stable in the serum, suggesting that the method was suitable for the analysis of a high number of serum samples.

**Table 4:** Stability of choline, betaine and TMAO standard solutions under different storage conditions. Experimental concentrations obtained after the storage time are expressed as µg g^**−**1^. Uncertainty of the values corresponds to the standard deviation of *n* = 3 independent replicates

Storage conditions	Before storage	After storage	% Recovery
	Betaine		
Short term (8 h at 10 °C) *n* = 3	0.373	0.385 ± 0.004	103 ± 1
	7.33	7.41 ± 0.05	102 ± 1
Short term (24 h at 25 °C) *n* = 5	0.354	0.346 ± 0.001	98 ± 1
	7.13	6.99 ± 0.03	96 ± 1
3 Freeze–thaw cycles (20 °C) *n* = 3	0.37	0.373 ± 0.002	102 ± 1
	7.27	7.12 ± 0.02	96 ± 1
	Choline		
Short term (8 h at 25 °C) *n* = 3	0.3232	0.3140 ± 0.0003	97 ± 1
	6.14	6.17 ± 0.01	101 ± 1
Short term (24 h at 25 °C) *n* = 5	0.346	0.323 ± 0.001	93 ± 1
	6.37	6.36 ± 0.01	100 ± 1
3 Freeze–thaw cycles (20 °C) *n* = 3	0.37	0.373 ± 0.001	101 ± 12
	6.60	6.99 ± 0.01	114 ± 1
	TMAO		
Short term (8 h at 25 °C) *n* = 3	0.1063	0.1147 ± 0.0002	108 ± 1
	2.06	2.066 ± 0.004	101 ± 1
Short term (24 h at 25 °C) *n* = 5	0.1066	0.1049 ± 0.0005	98 ± 1
	2.058	2.071 ± 0.009	101 ± 1
3 Freeze–thaw cycles (20 °C) *n* = 3	0.11	0.1050 ± 0.0002	99 ± 1
	2.05	2.060 ± 0.004	101 ± 1

#### Analysis of serum samples of patients with ischemic stroke

The optimized and validated 2D-HPLC–ESI–MS/MS method was applied to the analysis of 74 serum samples from patients who had suffered from an ischemic stroke in the past 24 h. A summary of the results obtained is given in Fig. S10. Also, a supplementary xls file with individual values is given as supporting material. Unfortunately, no control group of healthy individuals or patients without recent stroke was included. Such a comparator group would have strengthened the clinical interpretation of the biomarker levels observed and provided a clearer understanding of the potential diagnostic or prognostic utility of choline, betaine, and TMAO. It was observed [45] that lower circulating levels of TMAO were related with a better adherence to a mediterranean diet with higher vegetable and legume intake. The median values (interquartile ranges) obtained, expressed as μg g^−1^, were 1.89 (1.05–3.25), 3.55 (1.65–6.43) and 0.22 (0.053–0.605) for choline, betaine and TMAO, respectively. All samples were within the linear range studied for choline. However, in the case of betaine there were two samples, with 11.23 and 10.28 µg g^−1^ respectively, above the linear limit studied. In the case of TMAO, only one sample of 0.053 µg g^−1^ was below the minimum concentration of the linear range. For the three compounds, the detection and quantification limits of the method were below the concentration values of the samples. The median serum levels obtained for choline and TMAO in these patients were comparable to the data reported in previous publications [43, 44].

## Conclusions

We have developed a candidate reference procedure for the determination of choline pathway metabolites based on bidimensional chromatography and isotope dilution tandem mass spectrometry. So far, there is no reference procedure validated according to the CLSI guidelines for the determination of these metabolites in human serum. The strategy based on the use of bidimensional chromatography provides several advantages such as the reduction of the matrix effects in the ESI source, a sensitivity enhancement for the three analytes and the maximization of the sample throughput of the SCX column. However, the main limitation encountered in this procedure is the need for a complete transfer of the analyte and its labelled analogue when chromatographic isotope effects occur. This is particularly relevant when using multideuterated analogues as internal standards. The solution to this problem was achieved by increasing the volume transferred in the heart-cut or the use of ^13^C or ^15^N labelled analogues co-eluting with the corresponding natural abundance analytes. The method has shown satisfactory accuracy and precision to quantify more than 70 samples with a high degree of reliability as demonstrated in the validation according to the CLSI guidelines. Evaluating the results obtained in real samples more research must be carry out to establish a clear and direct relationship between TMAO levels and stroke prognosis despite lower TMAO levels demonstrate a good adherence to mediterranean diet.

## Supplementary Information

Below is the link to the electronic supplementary material.

Supplementary file1 (PDF 602 KB)

Supplementary file2 (XLSX 13 KB)

## Data Availability

All data are included in the paper and the Supporting Information and can be also available from the authors by request.

## References

[1] World Health Organization. Stroke, cerebrovascular accident [Internet]. Available from: https://www.emro.who.int/health-topics/stroke-cerebrovascular-accident/index.html. Accessed 16 May 2025.

[2] World Health Organization. World Stroke Day 2022. https://www.who.int/srilanka/news/detail/29-10-2022-world-stroke-day-2022. Accessed 16 May 2025.

[3] Zeisel SH, Blusztajn JK. Choline and human nutrition. Annu Rev Nutr. 1994;14(1):269–96. 10.1146/annurev.nu.14.070194.001413. 7946521 10.1146/annurev.nu.14.070194.001413

[4] Zeisel SH. Choline: critical role during fetal development and dietary requirements in adults. Annu Rev Nutr. 2006;26(1):229–50. 10.1146/annurev.nutr.26.061505.111156. 16848706 10.1146/annurev.nutr.26.061505.111156 PMC2441939

[5] Mi S, Zhao Y, Jacobs RL, Curtis JM. Simultaneous determination of trimethylamine and trimethylamine N-oxide in mouse plasma samples by hydrophilic interaction liquid chromatography coupled to tandem mass spectrometry. J Sep Sci. 2017;40(3):688–96. 10.1002/jssc.201600926. 27891771 10.1002/jssc.201600926

[6] Wortmann SB, Mayr JA. Choline-related-inherited metabolic diseases—a mini review. J Inherit Metab Dis. 2019;42(2):237–42. 10.1002/jimd.12011. 30681159 10.1002/jimd.12011 PMC7814885

[7] Zhong C, et al. Plasma choline and betaine and risks of cardiovascular events and recurrent stroke after ischemic stroke. Am J Clin Nutr. 2021;114(4):1351–9. 10.1093/ajcn/nqab199. 34159355 10.1093/ajcn/nqab199

[8] Zhong C, et al. Choline pathway nutrients and metabolites and cognitive impairment after acute ischemic stroke. Stroke. 2021;52(3):887–95. 10.1161/STROKEAHA.120.031903. 33467878 10.1161/STROKEAHA.120.031903

[9] Millard HR, et al. Dietary choline and betaine; associations with subclinical markers of cardiovascular disease risk and incidence of CVD, coronary heart disease and stroke: the Jackson Heart Study. Eur J Nutr. 2018;57(1):51–60. 10.1007/s00394-016-1296-8. 27550622 10.1007/s00394-016-1296-8 PMC5931705

[10] Xue J, et al. Residual risk of trimethylamine‐N‐oxide and choline for stroke recurrence in patients with intensive secondary therapy. J Am Heart Assoc. 2022;11(19). 10.1161/JAHA.122.027265. 10.1161/JAHA.122.027265 PMC9673713 36193936

[11] Xie L, et al. A U-shaped association between serum betaine and incident risk of first ischemic stroke in hypertensive patients. Clin Nutr. 2020;39(8):2517–24. 10.1016/j.clnu.2019.11.011. 31806397 10.1016/j.clnu.2019.11.011

[12] Liu D, Gu S, Zhou Z, Ma Z, Zuo H. Associations of plasma TMAO and its precursors with stroke risk in the general population: a nested case-control study. J Intern Med. 2023;293(1):110–20. 10.1111/joim.13572. 36200542 10.1111/joim.13572

[13] Chen Y-Y, Ye Z-S, Xia N-G, Xu Y. TMAO as a novel predictor of major adverse vascular events and recurrence in patients with large artery atherosclerotic ischemic stroke. Clin Appl Thromb Hemost. 2022;28:10760296221090504. 10.1177/10760296221090503. 35345908 10.1177/10760296221090503 PMC8969508

[14] Zhang J, et al. Gut microbial metabolite TMAO portends prognosis in acute ischemic stroke. J Neuroimmunol. 2021;354:577526. 10.1016/j.jneuroim.2021.577526. 33647820 10.1016/j.jneuroim.2021.577526

[15] Principles and Applications of Clinical Mass Spectrometry. Elsevier; 2018. 10.1016/C2017-0-03476-6.

[16] Jannetto PJ, Fitzgerald RL. Effective use of mass spectrometry in the clinical laboratory. Clin Chem. 2016;62(1):92–8. 10.1373/clinchem.2015.248146. 26553795 10.1373/clinchem.2015.248146

[17] Leung KS-Y, Fong BM-W. LC–MS/MS in the routine clinical laboratory: has its time come? Anal Bioanal Chem. 2014;406(9–10):2289–301. 10.1007/s00216-013-7542-5. 24337187 10.1007/s00216-013-7542-5

[18] Himmelsbach M. 10 years of MS instrumental developments – Impact on LC–MS/MS in clinical chemistry. J Chromatogr B. 2012;883–884:3–17. 10.1016/j.jchromb.2011.11.038. 10.1016/j.jchromb.2011.11.038 22177236

[19] Gosetti F, et al. Ultra high performance liquid chromatography tandem mass spectrometry determination and profiling of prohibited steroids in human biological matrices. J Chromatogr B. 2013;927:22–36. 10.1016/j.jchromb.2012.12.003. 10.1016/j.jchromb.2012.12.003 23317577

[20] Vogeser M, Seger C. Pitfalls associated with the use of liquid chromatography-tandem mass spectrometry in the clinical laboratory. Clin Chem. 2010;56(8):1234–44. 10.1373/clinchem.2009.138602. 20511452 10.1373/clinchem.2009.138602

[21] Teo TL, Boles JO, Munro K, Jones OA, Chai SY, Brown R, et al. Enhancing the accuracy of measurement of small molecule organic biomarkers. Anal Bioanal Chem. 2019;411(28):7341–55. 10.1007/s00216-019-02153-x. 31667564 10.1007/s00216-019-02153-x PMC11087866

[22] García Alonso JI, Rodriguez-González P. Isotope Dilution Mass Spectrometry. London: The Royal Society of Chemistry; 2013.

[23] De Bièvre P, Peiser HS. Basic equations and uncertainties in isotope-dilution mass spectrometry for traceability to SI of values obtained by this primary method. Fresenius J Anal Chem. 1997;359(7–8):523–5. 10.1007/s002160050625.

[24] Milton MJT, Quinn TJ. Primary methods for the measurement of amount of substance. Metrologia. 2001;38(4):289–96. 10.1088/0026-1394/38/4/1.

[25] Welch MJ, Breen JJ, Cohen A, Hertz HS, Ng LLY, Schaffer R, et al. Determination of serum urea by isotope dilution mass spectrometry as a candidate definitive method. Anal Chem. 1984;56(4):713–9. 10.1021/ac00268a028. 6372546 10.1021/ac00268a028

[26] Siekmann L. Determination of creatinine in human serum by isotope dilution-mass spectrometry. Definitive methods in clinical chemistry, IV. Clin Chem Lab Med. 1985;23(3):137. 10.1515/cclm.1985.23.3.137. 3889218

[27] Dodder NG, Tai SS-C, Sniegoski LT, Zhang NF, Welch MJ. Certification of creatinine in a human serum reference material by GC-MS and LC-MS. Clin Chem. 2007;53(9):1694–9. 10.1373/clinchem.2007.090027. 17660272 10.1373/clinchem.2007.090027

[28] Gradl K, Lattwein D, Karst U, Kranenburg RF, van der Linden N, Cobbaert C, et al. An isotope dilution LC–MS/MS-based candidate reference method for the quantification of androstenedione in human serum and plasma. Clin Mass Spectrom. 2020;16:1–10. 10.1016/j.clinms.2020.01.003. 34820514 10.1016/j.clinms.2020.01.003 PMC8600989

[29] Schierscher T, del Mar Rodríguez-González M, Petek M, van den Ouweland JMW, Vesper HW, Cobbaert CM, et al. An isotope dilution-liquid chromatography-tandem mass spectrometry (ID-LC-MS/MS)-based candidate reference measurement procedure for the quantification of zonisamide in human serum and plasma. Clin Chem Lab Med. 2023;0(0). 10.1515/cclm-2023-0736. 10.1515/cclm-2023-0736 38105272

[30] Stoyanov AV, Rohlfing CL, Connolly S, Roberts ML, Nauser CL, Little RR. Use of cation exchange chromatography for human C-peptide isotope dilution – Mass spectrometric assay. J Chromatogr A. 2011;1218(51):9244–9. 10.1016/j.chroma.2011.10.080. 22098929 10.1016/j.chroma.2011.10.080 PMC5089808

[31] Chen Y, Teo HL, Liu Q, Lee TK. Developing a reference measurement procedure for free glycerol in human serum by two-step gas chromatography–isotope dilution mass spectrometry. Clin Biochem. 2015;48(13–14):897–903. 10.1016/j.clinbiochem.2015.05.022. 26054581 10.1016/j.clinbiochem.2015.05.022

[32] Fernández AS, Rodríguez-González P, Álvarez L, García M, Iglesias HG, García Alonso JI. Multiple heart-cutting two-dimensional liquid chromatography and isotope dilution tandem mass spectrometry for the absolute quantification of proteins in human serum. Anal Chim Acta. 2021;1184:339022. 10.1016/j.aca.2021.339022. 34625263 10.1016/j.aca.2021.339022

[33] Giménez-Coral AC, Camacho-Muñoz D, Fernández-Ramos M, Cuadros-Rodríguez L, García-Campaña AM, Barranco A. Comparison between one and two-dimensional liquid chromatographic approaches for the determination of plasmatic stroke biomarkers by isotope dilution and tandem mass spectrometry. Analyst. 2023;148(3):583–93. 10.1039/D2AN01750D. 36594438 10.1039/d2an01750d

[34] Pineda-Cevallos D, Funes Menéndez M, González-Gago A, Rodríguez-González P, García Alonso JI. Correction of creatine-creatinine conversion during serum creatinine quantification by two-dimensional liquid chromatography and double-spike isotope dilution tandem mass spectrometry. Clin Chim Acta. 2024;554:117778. 10.1016/j.cca.2024.117778. 38220136 10.1016/j.cca.2024.117778

[35] Stoll DR, Carr PW. Two-dimensional liquid chromatography: a state-of-the-art tutorial. Anal Chem. 2017;89(1):519–31. 10.1021/acs.analchem.6b03506. 27935671 10.1021/acs.analchem.6b03506

[36] Pirok BWJ, Stoll DR, Schoenmakers PJ. Recent developments in two-dimensional liquid chromatography: fundamental improvements for practical applications. Anal Chem. 2019;91(1):240–63. 10.1021/acs.analchem.8b04841. 30380827 10.1021/acs.analchem.8b04841 PMC6322149

[37] Pursch M, Lewer P, Buckenmaier S. Resolving co-elution problems of components in complex mixtures by multiple heart-cutting 2D-LC. Chromatographia. 2017;80(1):31–8. 10.1007/s10337-016-3214-x.

[38] Hyung S, Kim B. Bias reduction in the quantitative analysis of a target analyte present in a limited quantity in human plasma using dual-mode heart-cutting two-dimensional liquid chromatography coupled with isotope dilution mass spectrometry. Biomed Chromatogr. 2020;34(7):e4831. 10.1002/bmc.4831. 32181511 10.1002/bmc.4831

[39] Ramaley L, Herrera LC. Software for the calculation of isotope patterns in tandem mass spectrometry. Rapid Commun Mass Spectrom. 2008;22(17):2707–14. 10.1002/rcm.3668. 18677719 10.1002/rcm.3668

[40] González-Antuña A, Rodríguez-González P, García Alonso JI. Determination of the enrichment of isotopically labelled molecules by mass spectrometry. J Mass Spectrom. 2014;49(8):681–91. 10.1002/jms.3397. 25044895 10.1002/jms.3397

[41] Fernández-Fernández M, Rodríguez-González P, AñónÁlvarez ME, Rodríguez F, Menéndez FVÁ, García Alonso JI. Simultaneous determination of creatinine and creatine in human serum by double-spike isotope dilution liquid chromatography–tandem mass spectrometry (LC-MS/MS) and gas chromatography–mass spectrometry (GC-MS). Anal Chem. 2015;87(7):3755–63. 10.1021/acs.analchem.5b00769. 25751287 10.1021/acs.analchem.5b00769

[42] Nie J, Lian FM, Yu J, Chen H, Yan X, Tang W, et al. Serum trimethylamine N-oxide concentration is positively associated with first stroke in hypertensive patients. Stroke. 2018;49(9):2021–8. 10.1161/STROKEAHA.118.021997. 30354996 10.1161/STROKEAHA.118.021997

[43] Amrein M, Rospleszcz S, Adam J, Koenig W, Meisinger C, Heier M, et al. Gut microbiota-dependent metabolite trimethylamine N-oxide (TMAO) and cardiovascular risk in patients with suspected functionally relevant coronary artery disease (fCAD). Clin Res Cardiol. 2022;111(6):692–704. 10.1007/s00392-022-01992-6. 35220448 10.1007/s00392-022-01992-6 PMC9151506

[44] Rexidamu M, Li H, Jin H, Huang J. Serum levels of trimethylamine-N-oxide in patients with ischemic stroke. Biosci Rep. 2019;39(6):BSR20190515. 10.1042/BSR20190515. 31142624 10.1042/BSR20190515 PMC6579976

[45] Castañón-Apilánez M, Ramiro-Bouza J, Bermejo-Pareja F, Oliva J, Villalobos F, Milla M, et al. Mediterranean diet prior to ischemic stroke and potential circulating mediators of favorable outcomes. Nutrients. 2024;16(18):3218. 10.3390/nu16183218. 39339817 10.3390/nu16183218 PMC11435288

