# Correction to: Development of a candidate reference method for the simultaneous quantification of betaine, choline and trimethylamine N‑oxide in serum samples by two‑dimensional liquid chromatography and isotope dilution tandem mass spectrometry

**DOI:** 10.1007/s00216-025-05960-7

**Published:** 2025-06-17

**Authors:** Daniela Pineda‑Cevallos, María Castañón Apilánez, Elena López-Cancio, Belén Prieto García, J. Ignacio García Alonso, Pablo Rodriguez‑Gonzalez

**Affiliations:** 1 Department of Physical and Analytical Chemistry, University of Oviedo, Avenida Julián Clavería, 8, 33006 Oviedo, Spain; 2 Department of Neurology, Central University Hospital of Asturias, 33011 Oviedo, Spain; 3 Clinical Biochemistry, Laboratory of Medicine, Central University Hospital of Asturias, 33011 Oviedo, Spain; 4 Instituto de Investigacion Sanitaria del Principado de Asturias (ISPA), 33011 Oviedo, Spain

**Correction to: Analytical and Bioanalytical Chemistry**

10.1007/s00216-025-05914-z

The original version of this article unfortunately contained a mistake.

One of the coauthors has reported a mistake in her name.

Elena López-Cancio is the right name instead of Elena Cancio‑López.

The original article has been corrected.

