# Supplementary material for: Development of a candidate reference method for the simultaneous quantification of betaine, choline and trimethylamine N-oxide in serum samples by two-dimensional liquid chromatography and isotope dilution tandem mass spectrometry
Source: Anal Bioanal Chem. 2025 May 23;417(18):4039–52. doi: 10.1007/s00216-025-05914-z (PMC12276144; doi:10.1007/s00216-025-05914-z)
Supplement: Supplementary file 1 — Supplementary file1 (PDF 602 KB) [file 216_2025_5914_MOESM1_ESM.pdf]

ELECTRONIC SUPPORING INFORMATION

DEVELOPMENT OF A CANDIDATE REFERENCE METHOD FOR THE SIMULTANEOUS QUANTIFICATION OF BETAINE, CHOLINE AND TRIMETHYLAMINE N-OXIDE IN SERUM SAMPLES BY TWO-DIMENSIONAL LIQUID CHROMATOGRAPHY AND ISOTOPE DILUTION TANDEM MASS SPECTROMETRY

[1,4]Daniela Pineda-Cevallos, [2,4]María Castañón Apilánez, [2,4]Elena Cancio-López, [3,4]Belén Prieto García, [1,4] J. Ignacio García Alonso, [1,4] Pablo Rodríguez-González*.

[1] Department of Physical and Analytical Chemistry, University of Oviedo, Avenida Julián Clavería, 8, 33006, Oviedo, Spain.
[2] Department of Neurology, Central University Hospital of Asturias, 33011 Oviedo, Spain
[3]Clinical Biochemistry, Laboratory of Medicine, Central University Hospital of Asturias, Oviedo, 33011, Spain
[4] Instituto de Investigación Sanitaria del Principado de Asturias (ISPA), 33011 Oviedo, Spain.
*Author for correspondence: rodriguezpablo@uniovi.es

Number of pages: 17

Number of Figures: 10

Number of Tables: 10

Table S1. Experimental conditions of the electrospray ionization source employed in this work.

2D-HPLC-MS/MS	
**Mass spectrometer**	Agilent 6460
**Ion source**	Electrospray jet stream
**Ionization mode**	Positive
**Gas temperature**	250 °C
**Gas flow**	7 L min^{-1}
**Sheath gas temperature**	400 °C
**Sheath gas flow**	12 L min^{-1}
**Nebulizer pressure**	25 psi
**Capillary voltage**	2500 V
**Nozzle voltage**	0 V

Table S2. Formula of precursor and product ions associated with the monitored SRM transitions for the analytes and labelled analogues applied in the 2D-HPLC-MS/MS method developed in this work.

Compound	Precursor ion	Product ion	SRM transition
**Choline**	$C_5H_{14}NO$	$C_3H_{10}N$	104→60<br>105→61
**Choline $^{13}C_1$**	$^{13}CC_4H_{14}NO$	$^{13}CC_2H_{10}N$	105→61<br>106→62
**Betaine**	$C_5H_{12}NO_2$	C_3H_8N	118→58<br>119→59
**Betaine D_{11}**	$C_5HD_{11}NO_2$	C_3D_8N	129→66<br>130→67
**TMAO**	$C_3H_{10}NO$	C_3H_8N	76→58<br>77→59
**TMAO $^{13}C_3$**	$^{13}C_3H_{10}NO$	$^{13}C_3H_8N$	78→60<br>79→61
**TMAO D_9**	C_3HD_9NO	C_3D_8N	85→66<br>86→67
**TMAO D_{18}**	$C_6HD_{18}N_2O_2$	C_3HD_9NO	169→85<br>170→86
**Dimer-TMAO**	$C_6H_{19}N_2O_2$	$C_3H_{10}NO$	151→76<br>152→77
**Dimer-TMAO $^{13}C_3$**	$^{13}C_6H_{19}N_2O_2$	$^{13}C_3H_{10}NO$	156→68<br>157→79

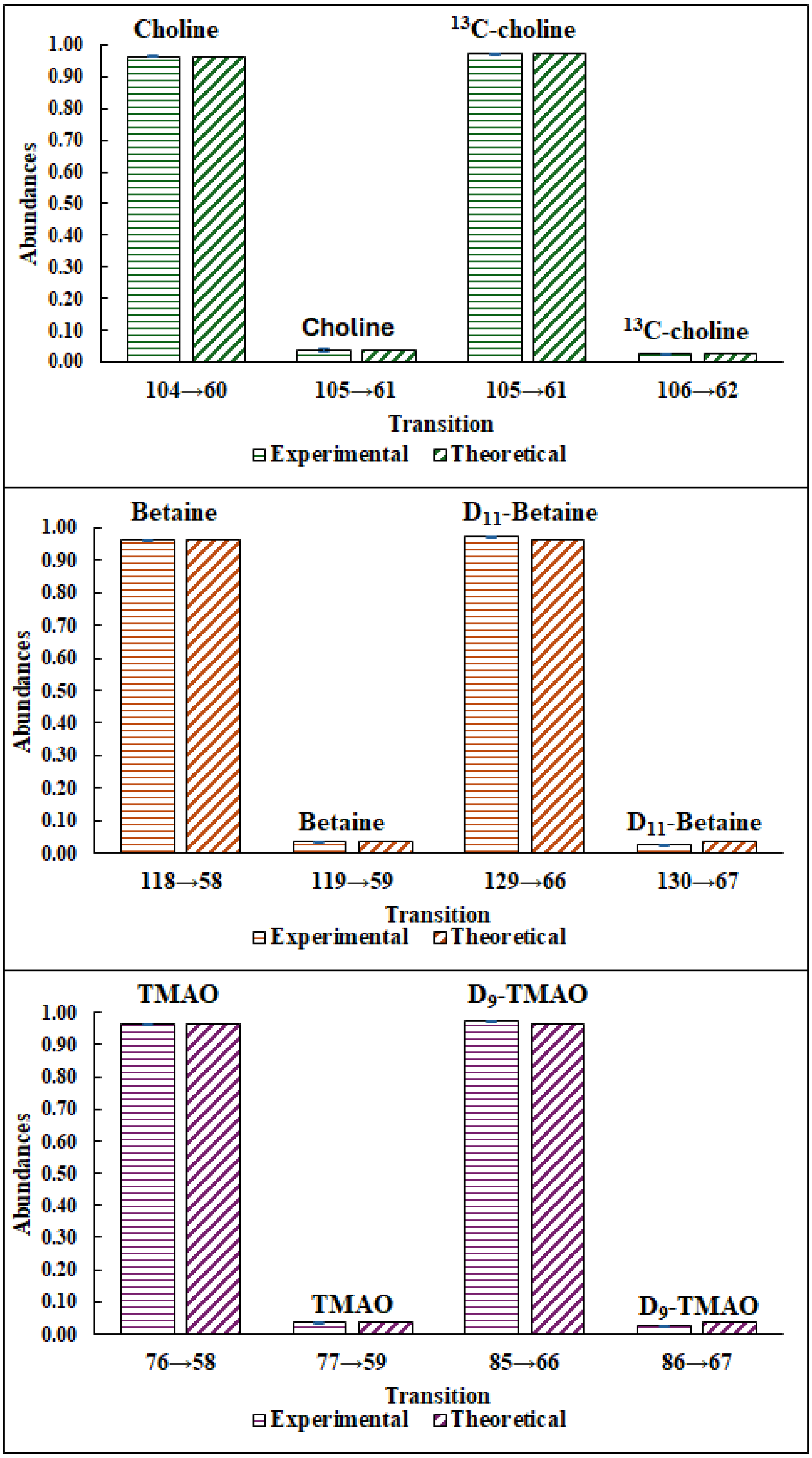

Figure S1. Comparison of theoretical and experimental isotopologue distributions for choline, betaine and TMAO. Experimental values were obtained by 2D-HPLC-ESI-MS/MS measurement for selected MRM transitions. Theoretical values were obtained using the specific dedicated software IsoPatrn© [1]. Error bars of the experimental values correspond to the standard deviation of n=5 independent injections.

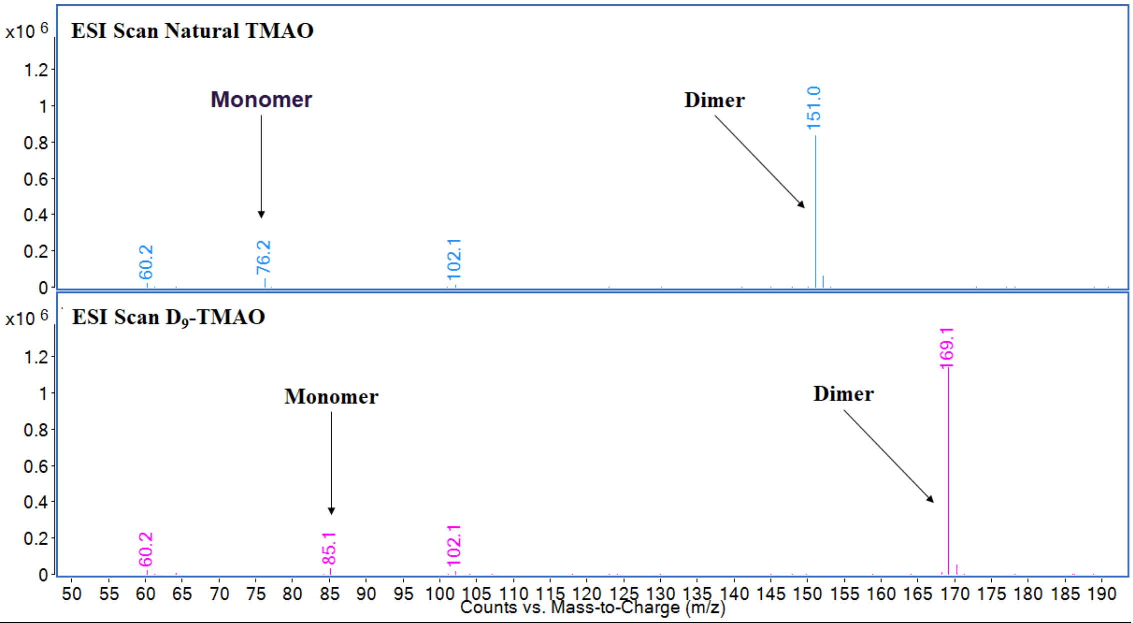

Figure S2. Mass spectrum of natural TMAO and labelled D_9-TMAO analogue obtained in the SCAN mode by LC-ESI-MS/MS.

A

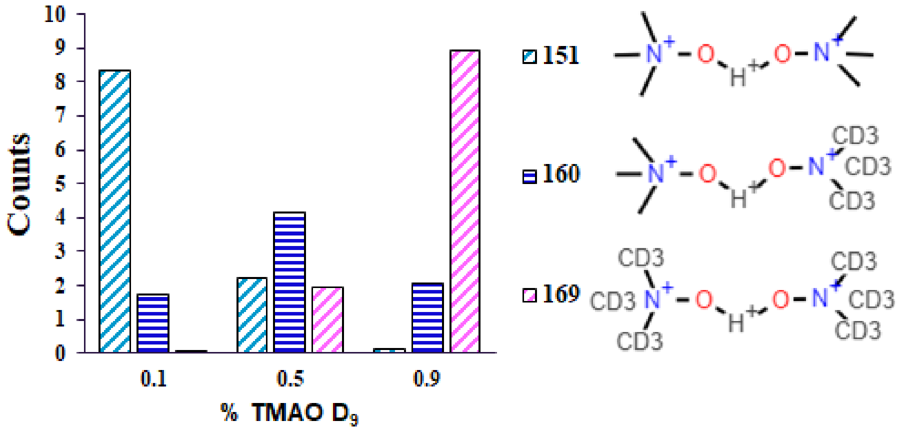

B

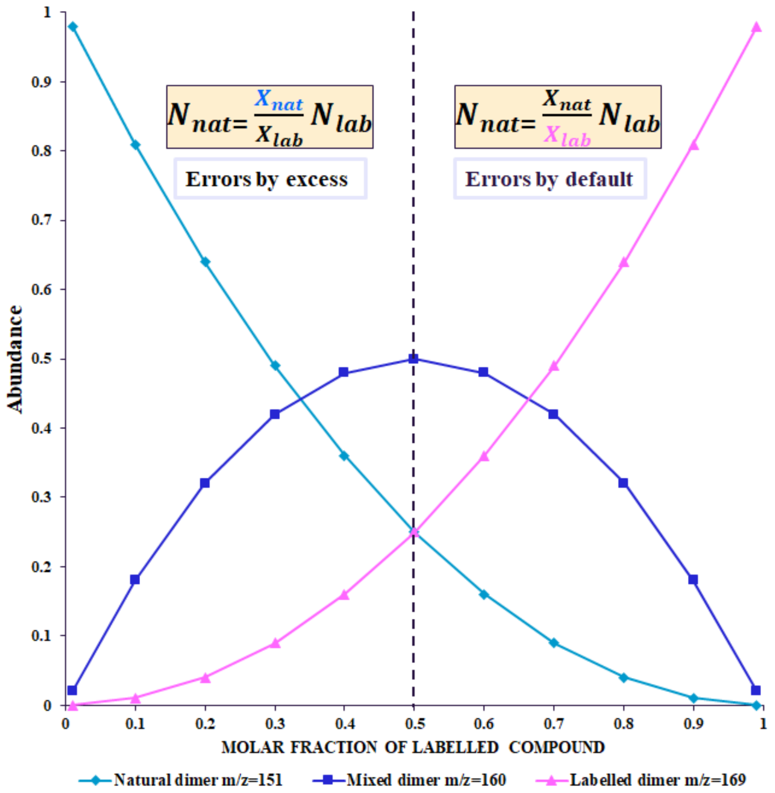

Figure S3. A) Evaluation of the TMAO dimer formation by 1D-HPLC-MS/MS analysis of a standard mixture of natural abundance TMAO and its labelled compound D_9-TMAO. Signal counts for natural (m/z=151), mixed (m/z=160) and labelled (m/z=169) dimer at different molar fraction of D_9-TMAO are shown **B)** Binomial distribution of TMAO and labelled analogue dimer formation. Abundance for natural (m/z=151), mixed (m/z=160) and labelled (m/z=169) dimer at different molar fractions of D_9-TMAO are shown.

Table S3. Theoretical concentration, experimental concentration and recovery (%) obtained for TMAO obtained in the analysis of standard solutions at three different levels by 1D-LC-MSMS adding the same amount of the labelled analogue. Analyses were performed using D_9-TMAO and $^{13}C_3$-TMAO as labelled analogues and monitoring the SRM transitions corresponding to the TMAO monomer and those of the TMAO dimer formed during ESI ionization. Uncertainty of the values corresponds to the standard deviation obtained from n=5 independent 1D-LC-MS/MS injections.

Deuterated D_9-TMAO					
		Monomer (85->66/86->67)		Dimer (169->85/170->86)	
X_{lab}	Theoretical (μg g^{-1})	Experimental (μg g^{-1})	% Recovery	Experimental (μg g^{-1})	% Recovery
0.70	0.110±0.004	0.1155±0.0003	112.0±0.3	0.0245±0.0001	23.8±0.1
0.20	1.087±0.008	1.084±0.004	99.7±0.4	2.13±0.01	196.2±1.2
0.10	2.07±0.01	2.09±0.01	101.8±1.3	8.2±0.1	720±13
^{13}C-TMAO					
		Monomer (78->60/79->61)		Dimer (156->78/157->79)	
X_{lab}	Theoretical (μg g-1)	Experimental (μg g-1)	%Recovery	Experimental (μg g-1)	% Recovery
0.70	0.110±0.004	0.1043±0.0004	101.2.0±0.4	0.0187±0.0001	18.13±0.05
0.20	1.087±0.008	1.095±0.003	100.7±0.3	2.114±0.005	194.4±0.4
0.10	2.07±0.01	2.139±0.008	107.0±0.8	7.72±0.03	675±3

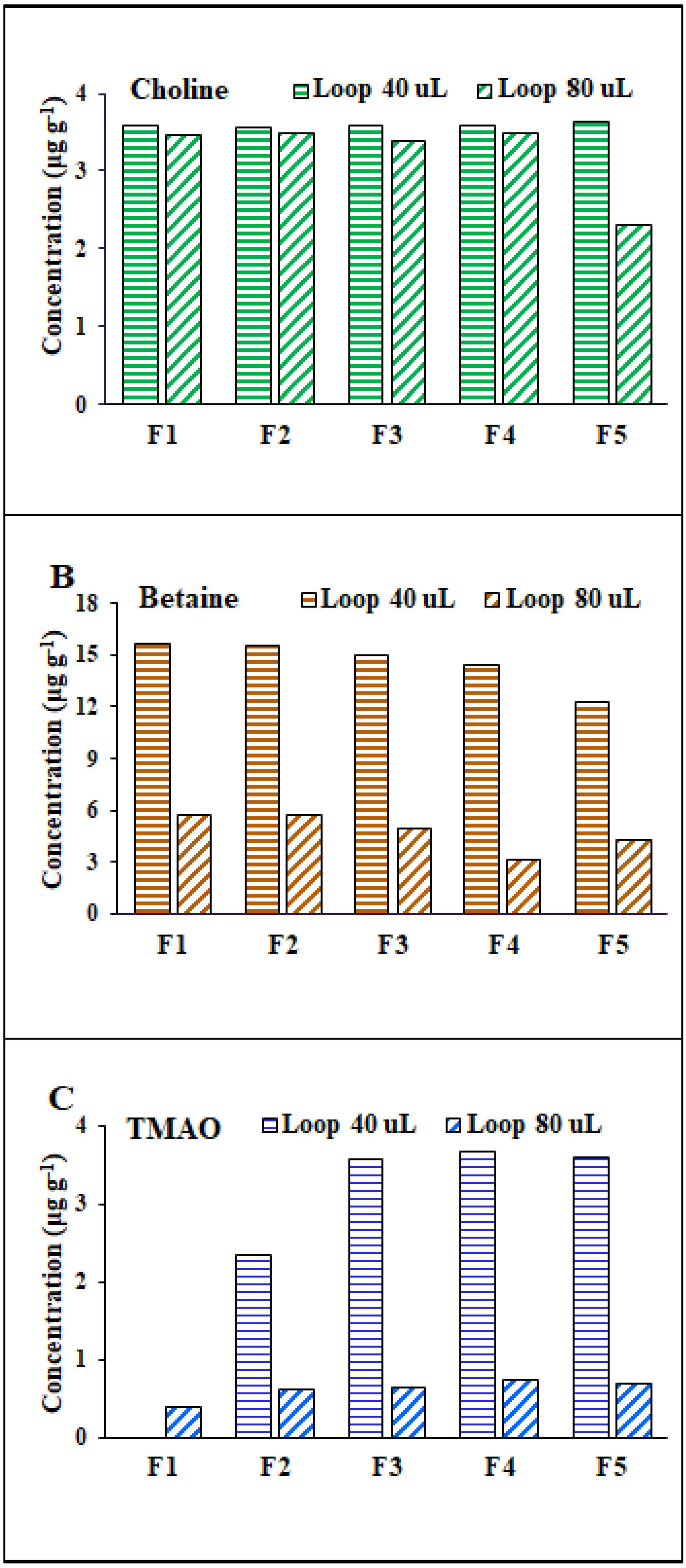

Figure S4. Concentrations for A) choline, B) betaine and C) TMAO in five consecutive heart-cuts of 40 µL and 80 µL transferred to the second dimension of a pooled serum sample analyzed by high resolution sampling and IDMS using $^{13}C_1$-choline, D_{11}-betaine and D_9-TMAO as labelled analogues.

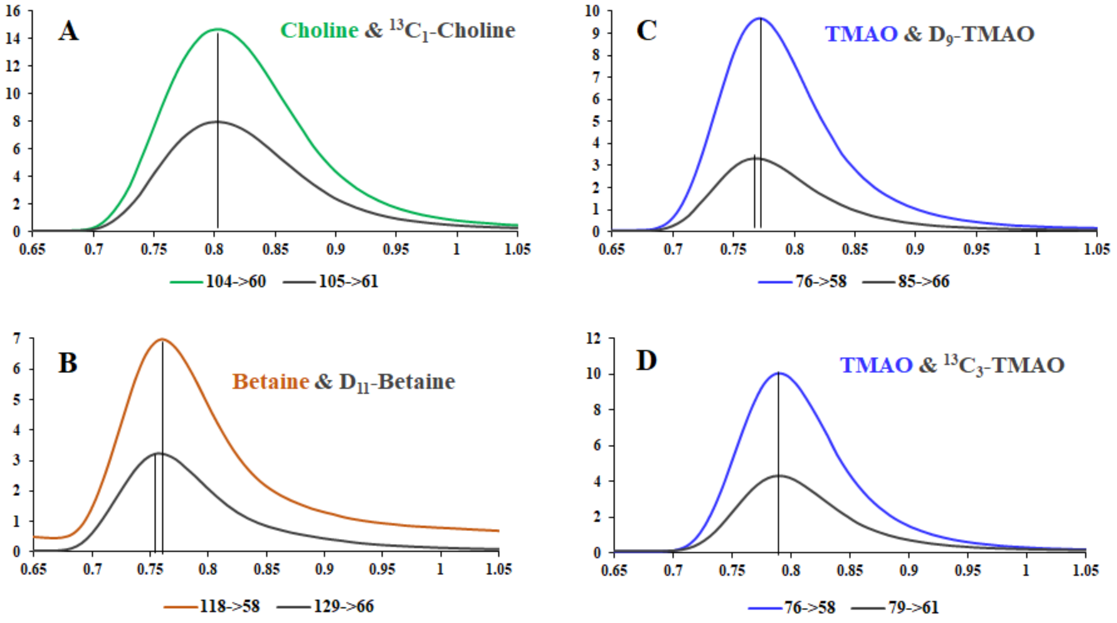

Figure S5. 1D-HPLC-MS/MS reverse phase MRM chromatograms of natural and labelled analogues for A) Choline B) Betaine and C) and D) TMAO to study their coelution in the first dimension. In the case of TMAO two different analogues are evaluated: $^{13}C_3$-TMAO and D_9-TMAO.

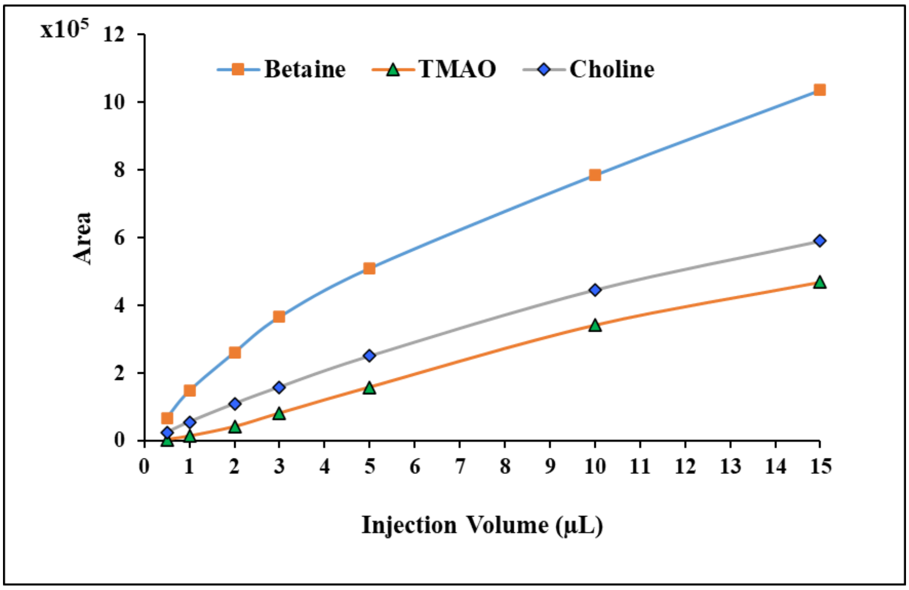

Figure S6. Choline, Betaine and TMAO peak area obtained in a pooled serum sample by 2D-LC-MS/MS at different injection volumes from 0.5 to 15 µL using a coeluting ^{13}C minimally labelled analogue for choline and deuterated analogues for both betaine and TMAO.

Table S4. Concentration (μg g^{-1}) levels of natural and labelled e compounds employed in the linearity assessment carried out in this work. Uncertainty of the values corresponds to the standard deviation of the concentration obtained for n=5 replicates.

Level	Choline	Choline $^{13}C_1$	Betaine	Betaine D_{11}	TMAO	TMAO D_9
N1	0.184±0.001	2.34	0.191±0.001	3.30	0.0588±0.0001	0.88
N2	0.2287±0.0009	2.34	0.250±0.001	3.30	0.0755±0.0005	0.88
N3	0.329±0.001	2.34	0.370±0.002	3.30	0.108±0.001	0.88
N4	0.667±0.003	2.34	0.769±0.001	3.30	0.2246±0.0007	0.88
N5	1.671±0.003	2.34	1.920±0.005	3.30	0.565±0.002	0.88
N0	3.292±0.007	2.34	3.748±0.004	3.30	1.103±0.002	0.88
N6	4.05±0.01	2.34	4.57±0.02	3.30	1.348±0.002	0.88
N7	6.21±0.03	2.34	7.17±0.03	3.30	2.087±0.003	0.88
N8	7.80±0.0.03	2.34	8.92±0.02	3.30	2.626±0.008	0.88

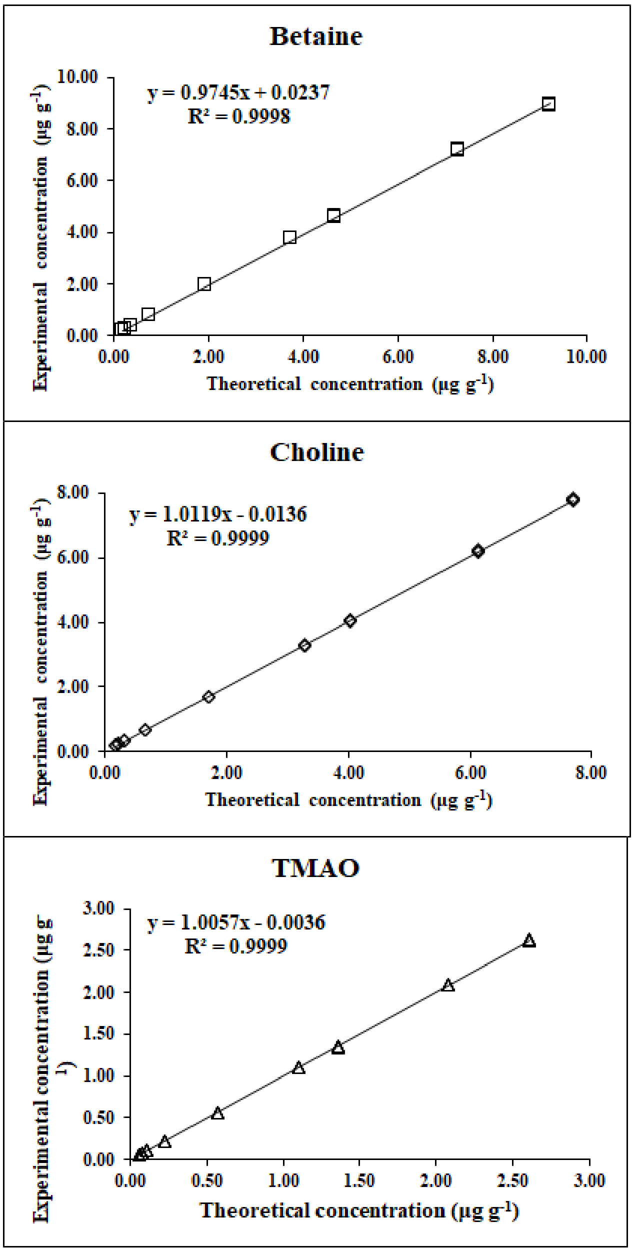

Figure S7. Correlation of the theoretical and experimental concentrations obtained in the linearity study of the method at nine concentration levels of choline, betaine and TMAO.

Table S5. Theoretical concentration, experimental concentration and recovery for choline in a diluted and fortified pooled serum with a known amount of the natural abundance analytes to achieve 5 different concentration levels measured by 2D-HPLC-MS/MS. This recovery experiment was carried out in six different measurement days. Uncertainty of the values corresponds to the standard deviation obtained from n=5 independent injections.

Level	Measurement day	Theoretical concentration (µg g^{-1})	Experimental concentration (µg g^{-1})	% Recovery
N1	1	0.17	0.184±0.001	108.7±0.7
	2	0.18	0.193±0.001	109.7±0.06
	3	0.17	0.1687±0.0004	96.0±0.2
	4	0.18	0.1962±0.0003	108.1±0.2
	5	0.18	0.1687±0.0004	96.0±0.2
	6	0.19	0.1775±0.0005	91.9±0.3
N3	1	0.32	0.329±0.001	101.8±0.3
	2	0.34	0.347±0.001	103.4±0.4
	3	0.32	0.315±0.002	97.5±0.5
	4	0.35	0.3206±0.0005	92.6±0.1
	5	0.34	0.322±0.001	96.1±0.4
	6	0.37	0.349±0.001	94.8±0.4
N6	1	4.04	4.05±0.01	101±1
	2	4.17	4.17±0.01	99±1
	3	4.05	4.06±0.02	102±2
	4	4.28	4.11±0.01	77.1±0.9
	5	4.17	4.15±0.01	97±1
	6	4.51	4.63±0.01	116.6±0.9
N7	1	6.13	6.21±0.03	103±1
	2	6.27	6.35±0.03	103±1
	3	6.14	6.17±0.01	100.8±0.5
	4	6.37	6.34±0.01	98.6±0.5
	5	6.26	6.04±0.02	92.3±0.6
	6	6.60	6.89±0.01	110.0±0.5
N8	1	7.70	7.80±0.03	102.2±0.8
	2	7.83	7.97±0.05	103±1
	3	7.71	7.70±0.04	99.9±0.9
	4	7.94	7.78±0.02	96.4±0.5
	5	7.83	7.63±0.04	95±1
	6	8.17	8.60±0.08	110±2

Table S6. Theoretical concentration, experimental concentration and recovery for betaine in a diluted and fortified pooled serum with a known amount of the natural abundance analytes to achieve 5 different concentration levels measured by 2D-HPLC-MS/MS. This recovery experiment was carried out in six different measurement days. Uncertainty of the values corresponds to the standard deviation obtained from n=5 independent injections.

Level	Measurement day	Theoretical concentration (µg g^{-1})	Experimental concentration (µg g^{-1})	% Recovery
N1	1	0.22	0.211±0.002	96.7±0.7
	2	0.19	0.202±0.002	104.8±0.8
	3	0.20	0.190±0.001	97.2±0.4
	4	0.19	0.1810±0.0004	97.7±0.2
	5	0.19	0.1890±0.0003	98.7±0.2
	6	0.19	0.191±0.001	99.4±0.7
N3	1	0.42	0.390±0.001	93.6±0.3
	2	0.37	0.365±0.001	99.4±0.3
	3	0.37	0.381±0.004	102±1
	4	0.35	0.350±0.002	98.9±0.5
	5	0.37	0.373±0.001	102.1±0.4
	6	0.37	0.370±0.002	100.7±0.4
N6	1	5.18	5.14±0.04	96±4
	2	4.68	4.49±0.01	79±1
	3	4.74	4.72±0.03	98±4
	4	4.54	4.49±0.01	95.2±0.9
	5	4.65	4.66±0.05	100±5
	6	4.68	4.57±0.02	88±2
N7	1	7.77	8.05±0.07	108±2
	2	7.27	7.18±0.10	97±3
	3	7.33	7.32±0.02	99.9±0.5
	4	7.13	7.03±0.01	97.2±0.3
	5	7.25	7.28±0.04	101±1
	6	7.27	7.17±0.03	97.2±0.8
N8	1	9.71	10±0.1	105±2
	2	9.21	9.1±0.3	99±6
	3	9.27	9.48±0.02	104.0±0.4
	4	9.07	9.1±0.1	100±3
	5	9.19	9.5±0.1	106±2
	6	9.21	8.92±0.02	94.8±0.4

Table S7. Theoretical concentration, experimental concentration and recovery for TMAO in a diluted and fortified pooled serum with a known amount of the natural abundance analytes to achieve 5 different concentration levels measured by 2D-HPLC-MS/MS. This recovery experiment was carried out in six different days. Uncertainty of the values corresponds to the standard deviation obtained from n=5 independent injections.

Level	Measurement day	Theoretical concentration (μg g^{-1})	Experimental concentration (μg g^{-1})	% Recovery
N1	1	0.06	0.0588±0.0001	103.8±0.2
	2	0.06	0.0575±0.0006	103±2
	3	0.06	0.0578±0.0002	103.7±0.3
	4	0.06	0.0579±0.0006	104±1
	5	0.06	0.0574±0.0006	103±1
	6	0.06	0.0562±0.0003	101.2±0.6
N3	1	0.11	0.1088±0.001	100±1
	2	0.11	0.115±0.001	108±1
	3	0.11	0.115±0.001	108.0±0.5
	4	0.11	0.107±0.001	100.5±0.5
	5	0.11	0.1074±0.0003	101.1±0.3
	6	0.11	0.1055±0.0005	99.6±0.5
N6	1	1.36	1.348±0.002	95.7±0.8
	2	1.34	1.341±0.004	100±1
	3	1.34	1.346±0.002	102±1
	4	1.34	1.349±0.003	102±1
	5	1.34	1.350±0.002	104.1±0.9
	6	1.34	1.334±0.003	99±1
N7	1	2.07	2.087±0.003	101.4±0.3
	2	2.06	2.074±0.004	101.9±0.4
	3	2.06	2.068±0.003	101.3±0.3
	4	2.06	2.075±0.005	101.7±0.5
	5	2.05	2.084±0.004	103.0±0.5
	6	2.05	2.053±0.003	100.2±0.3
N8	1	2.61	2.626±0.008	102.2±0.8
	2	2.59	2.622±0.008	102.1±0.6
	3	2.59	2.630±0.003	102.7±0.2
	4	2.59	2.623±0.006	101.9±0.4
	5	2.59	2.639±0.005	103.3±0.3
	6	2.59	2.594±0.004	100.5±0.3

Table S8 Average concentration, standard deviation (SD), limit of the blank (LoB), limit of detection (LoD) and limit of quantification (LoQ) calculated from the measurements of blanks (B) and low concentration samples (LCS) for choline, betaine and TMAO.

		Replicates	Average (μg g^{-1})	SD	LoB	LoD	LoQ
**Choline**	B	60	0.02	0.01	0.03		0.08
	LCS	72	0.18	0.01		0.04	0.07
**Betaine**	B	60	0.01	0.01	0.03		0.12
	LCS	72	0.20	0.01		0.05	0.14
**TMAO**	B	60	0.003	0.005	0.01		0.05
	LCS	72	0.057	0.001		0.01	0.01

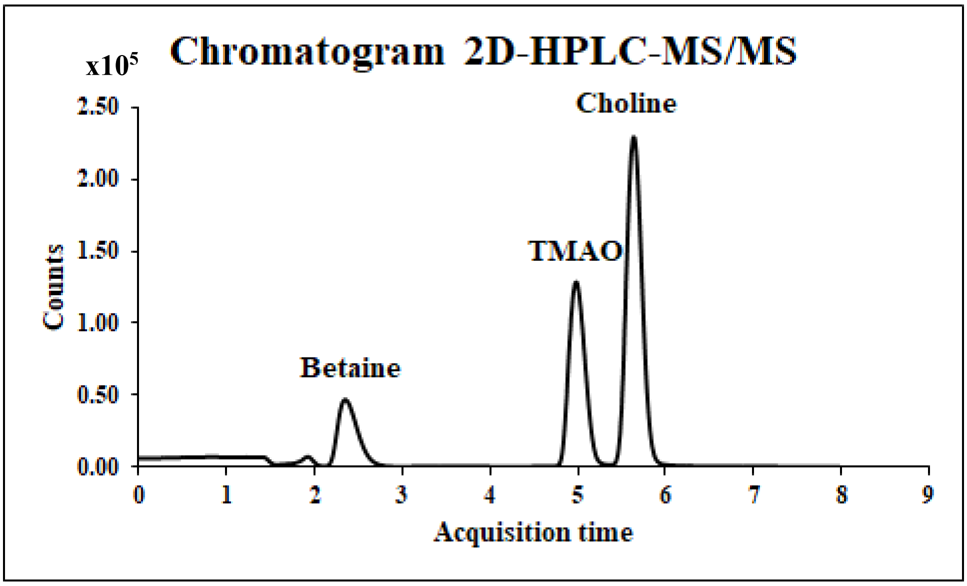

Figure S8. 2D-HPLC-MS/MS chromatogram (total ion count) of a 1:20 dilution of a human pooled serum with PBS containing 0.20 μg g^{-1} of betaine, 0.18 μg g^{-1} of choline and 0.057 μg g^{-1} of TMAO and spiked with 0.23 μg g^{-1} of D_{11} betaine, 0.15 μg g^{-1} of $^{13}C_1$ choline and 0.056 μg g^{-1} of D_9 TMAO.

Table S9 Concentration (μg g^{-1}), % Recovery, % CV and % accuracy of choline, betaine and TMAO in quality control samples. Uncertainty of the values corresponds to the standard deviation of the concentration obtained for the indicated replicates.

	Choline		
**Measurement day**	**Concentration (μg g^{-1}) n=3**	**%Recovery**	**%CV**
1	2.017±0.002	101.6±0.1	0.08
2	2.002±0.003	100.8±0.2	0.16
3	1.986±0.005	100.0±0.3	0.27
4	1.876±0.004	94.5±0.2	0.22
5	1.836±0.002	92.4±0.1	0.11
6	2.200±0.002	110.8±0.1	0.11
Theoretical Concentration (μg g^{-1})	1.986		
**Average (n=18)**	**1.986±0.128**	**100.0±6.4**	**6.43**
**Measurement day**	**Betaine**		
1	2.65±0.02	107.7±0.8	0.75
2	2.50±0.02	101.5±0.9	0.92
3	2.44±0.01	99.1±0.6	0.61
4	2.14±0.03	87.2±1.4	1.58
5	2.27±0.09	92.2±3.6	3.91
6	2.32±0.08	94.2±3.2	3.40
Theoretical concentration (μg g^{-1})	2.46		
**Average (n=18)**	**2.39±0.18**	**97.0±7.3**	**7.53**
**Measurement day**	**TMAO**		
1	0.686±0.003	101.2±0.5	0.48
2	0.680±0.001	100.2±0.2	0.17
3	0.681±0.002	100.4±0.3	0.27
4	0.713±0.000	105.1±0.0	0.02
5	0.684±0.006	100.8±0.9	0.92
6	0.678±0.001	100.0±0.2	0.20
Theoretical Concentration (μg g^{-1})	0.678		
**Average (n=18)**	**0.687±0.013**	**101.3±1.9**	**1.88**

Table S10. Summary data from Fischer test two-sample for variances and t-test assuming unequal variances. Low concentration samples were injected after measurement of 50 times more concentrated samples (HCS) versus sequentially injected low concentration samples for choline, betaine and TMAO for carryover evaluation.

	Choline		Betaine		TMAO	
	after HCS	Sequential	after HCS	Sequential	after HCS	Sequential
**Mean µg g^{-1}**	0.182	0.178	0.191	0.186	0.057	0.057
**Variance**	9.00E-05	4.74E-07	8.14E-05	5.38E-07	1.39E-06	1.68E-07
**n**	11	11	12	12	12	12
**F**	190.06		151.27		8.29	
**P(F<=f)** $_{\text{one-tail}}$	4.86E-10		2.32E-10		7.44E-04	
**F** $_{\text{Critical one-tail}}$	2.98		2.82		2.82	
**t** $_{\text{Stat}}$	1.26		1.97		-0.83	
**P(T<=t)** $_{\text{two-tail}}$	0.24		0.07		0.42	
**t** $_{\text{Critical two-tail}}$	2.23		2.20		2.14	

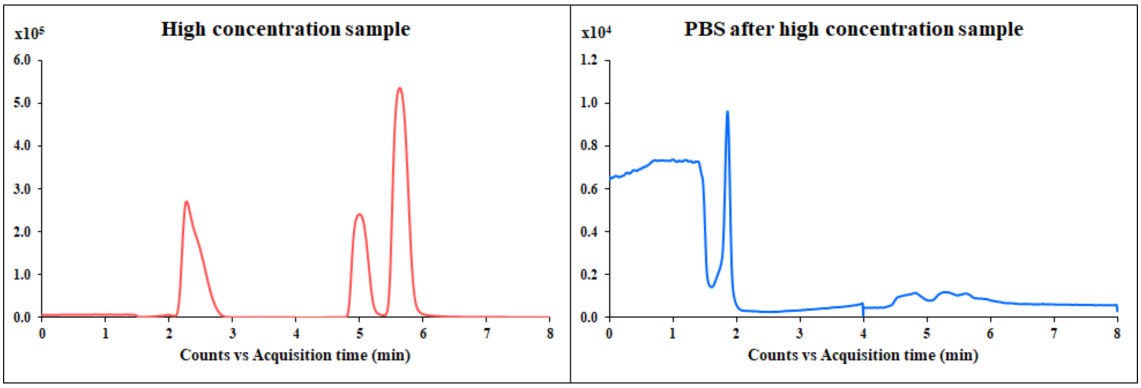

Figure S9. 2D-HPLC-MS/MS chromatogram of a high concentration pool serum sample that contains 9.24, 2.62 and 7.78 µg g^{-1} of betaine, TMAO and choline, respectively vs a chromatogram of PBS injected after the high concentrated sample. No detectable signal of the analytes was observed.

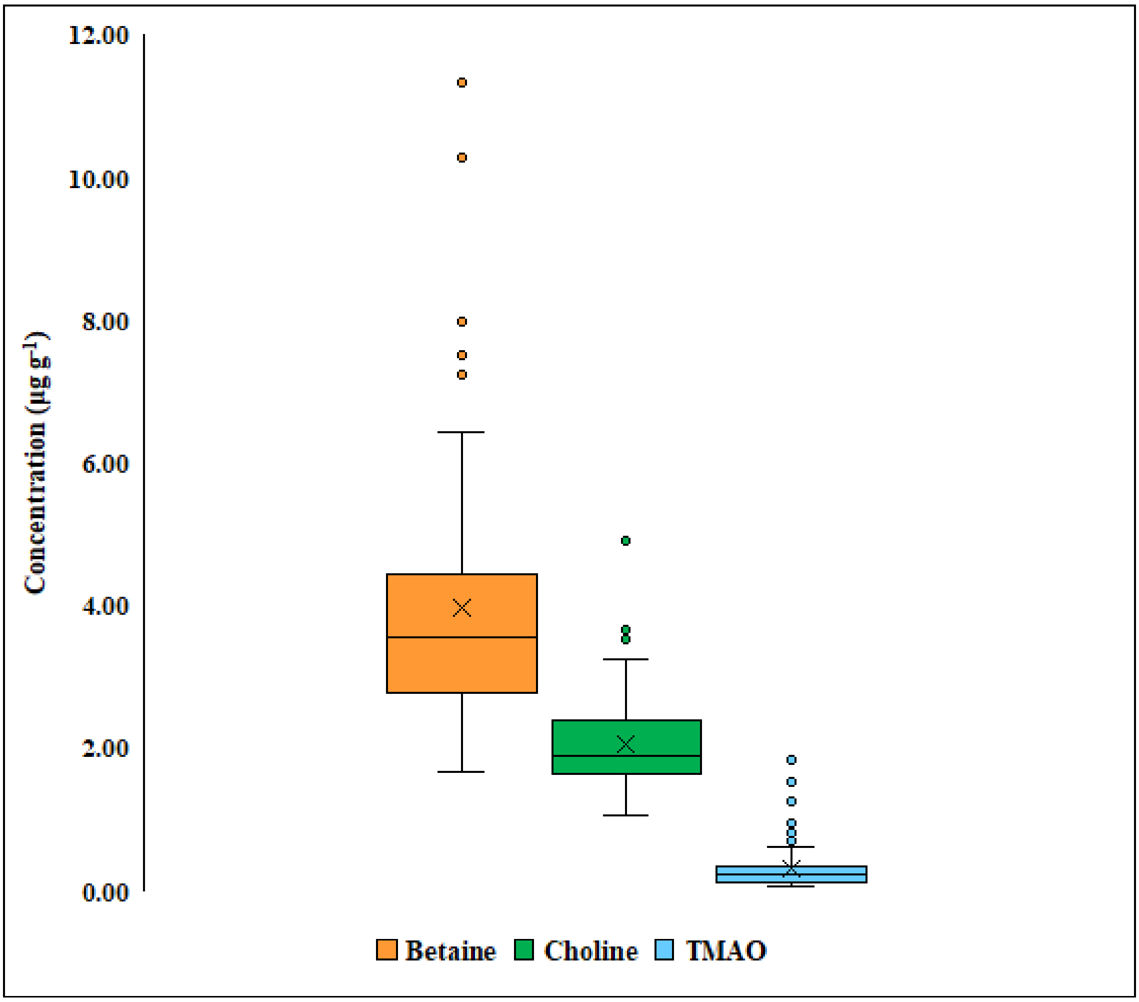

Figure S10. Summary of the results obtained in the analysis of 74 serum samples from patients who had suffered from an ischemic stroke in the past 24 hours

REFERENCES.

[1] Ramaley L, Herrera LC. Software for the calculation of isotope patterns in tandem mass spectrometry. Rapid Commun Mass Spectrom. 2008 Sep;22(17):2707–14. doi: 10.1002/rcm.3668
